# Molecular docking, ADMET profiling of gallic acid and its derivatives (N-alkyl gallamide) as an anti-breast cancer agent

**DOI:** 10.12688/f1000research.127347.1

**Published:** 2022-12-08

**Authors:** Ade Arsianti, Norma Nur Azizah, Linda Erlina

**Affiliations:** 1 Master’s Programme in Biomedical Science, Faculty of Medicine, Universitas Indonesia, Jakarta, 10360, Indonesia; 2 Drug Development Research Center, Indonesian Medical Education and Research Institute (IMERI), Jakarta, 10630, Indonesia; 3 Department of Medical Chemistry, Faculty of Medicine, Universitas Indonesia, Jakarta, 10360, Indonesia; 4 Bioinformatics Core Facilities, Indonesian Medical Education and Research Institute (IMERI), Jakarta, 10630, Indonesia

**Keywords:** N-Alkyl gallamide, molecular docking, ADMET, breast cancer

## Abstract

Background

In 2020, breast cancer has become the most common cancer in the world and in Indonesia. Searching for anticancer drugs using computational methods is considered more effective and selective than other methods. Gallic acid and its derivatives (esters and amides) are compounds that have biological activities such as anticancer effects. The purpose of this study was to analyse the molecular modelling and ADMET (Adsorption, Distribution, Metabolism, Excretion and Toxicity) profile of gallic acid derivative compounds (N-alkyl gallamides) as anticancer agents.

Methods

Target proteins were selected by analysis of protein-protein and drug-protein interactions. Molecular modelling was done by molecular docking. Predictive analysis of the ADMET profile of gallic acid and its derivatives (N-alkyl gallamide) was conducted using Marvin Sketch, Swissadme, protox II, and pkCSM pharmacokinetics. The selected target proteins were JUN, AKT1, CASP3, and CASP7.

Results

Compounds N-octyl gallamide, N-ters-butyl gallamide, and N-isoamil gallamide were the three best gallic acid derivatives based on molecular modelling analysis of target proteins associated with breast cancer. The ADMET profile of the N-alkyl gallamide compound is predictable and shows a good profile as a candidate for anticancer drugs.

Conclusion

N-octyl gallamide, N-ters-butyl gallamide, and N-isoamil gallamide have potential as anti-breast cancer agents.

## Introduction

Cancer is ranked second globally in diseases that could lead to death and caused 10 million deaths in 2020. Generally, the most prevalent types of cancer are breast, colorectal, and lung cancers. ^1^^,^^2^ Based on *Global Cancer Observatory* (GLOBOCAN), cancer cases in Indonesia increased in 2020, reaching 396,914 cases with a total death toll of 234,511. The leading forms of cancer were breast and cervical cancers, with the number of cases being 65,858 (16,6%) and 36,633 (9,2%), respectively. ^3^

Over recent years, research on anticancer therapies has been increasing, using various materials as anticancer drugs, which could be obtained via synthetic methods. ^4^^–^^6^ Although there are many potential anticancer drugs that have been synthesized, the medical needs are far from fulfilled. Various factors could affect the insufficiency of these drugs to meet these needs, including selectivity limitations of conventional drugs that could lead to toxicity, as well as the presence of metastasis and multi-drug resistance. ^7^^,^^8^ The search for selective and effective anticancer agents can be improved by computational methods. Computational methods have been used to significantly accelerate the drug design process. Protein-protein interactions, drug-protein interactions, and molecular modeling are the easiest methods to find and predict new molecules as potential drugs with shorter time and lower costs than conventional methods. ^9^

Computer-based molecular modelling merges informatics methods, medical sciences, and biophysics. The combination of these fields could predict the efficiency of potential therapeutic molecules that are designed before *in vitro* and pre-clinical tests. ^10^ Characteristics and profiles of adsorption, distribution, metabolism, excretion, and toxicity (ADMET), become one of the important parameters in the discovery and development of drug candidates to determine the feasibility of drug candidates before being developed in the next stage. ^10^

In recent decades, gallic acid has become a compound that is in great demand as an anticancer therapeutic agent. Gallic acid (3,4,5-trihydroxybenzoic acid) is widely contained in natural ingredients which has been reported to have antioxidant, antifungal, antiviral, anti-inflammatory, and anticancer activities. ^11^ Gallic acid has also been reported to have biological activity against several cancer cell lines such as leukemia, lung cancer, colorectal cancer, and breast cancer cell lines and does not show biological activity against normal lymphocyte cells. ^12^^,^^13^

According to Silva *et al*., (2017), gallic acid and its derivatives (alkyl esters) have pro-apoptotic activity and show as non-genotoxic and non-mutagenic agents to prevent chromosomal damage due to chemical exposure. ^14^ However, because gallic acid is a hydrophilic compound, it is more difficult for it to penetrate the hydrophobic cell membrane. Dorothea *et al*., (2016) concluded that increasing the potential for cytotoxicity to MCF7 breast cancer cells, lipophilicity must be achieved by modifying the structure of gallic acid by adding a lipophilic group to make it more hydrophobic. ^15^ According to in silico study, gallic acid and its derivatives (alkyl ester) can be BRAF inhibitor of colorectal cancer, ^16^ as well as inhibitor of the anti-apoptotic protein Bcl-xL in breast cancer. ^17^^,^^18^ In addition, gallic acid derivative compounds (N-alkyl gallamide) have strong cytotoxic activity on MCF7 breast cancer cells, and colorectal cancer cells, HCT 116. ^19^^,^^20^ This study aims to design gallic acid derivative compounds (N-alkyl Gallamide), analyze molecular modeling of target proteins associated with breast cancer, and determine the ADMET profile of these compounds as anti-breast cancer agents.

## Methods

### Protein data collection

Protein or gene data that are related to the incidence of breast cancer were downloaded from the KEGG (Kyoto Encyclopedia of Genes and Genome) database with keywords human diseases and breast cancer. In addition, apoptosis-related genes were searched and downloaded. The list of gene names was downloaded by copying all the genes into a Microsoft Excel (RRID:SCR_016137) sheet. The list of gene names is shown in repository data with accession number S-BSST934. ^21^

### Drug-protein interactions

The list of genes or proteins that were downloaded were then analyzed for interactions between gallic acid as a drug and a list of genes associated with breast cancer and apoptosis as target proteins. This analysis used a website-based tool called STITCH ( http://stitch.embl.de/). The list of genes and gallic acid was uploaded to the STITCH webserver with the search multiple names parameter, and the selected organism as *Homo sapiens.* The confidence score was also regulated in this analysis. The selected confidence score was the high confidence category (0.700). The interaction results were later downloaded in the TSV format. Visualization of drug-protein interactions was performed using Cytoscape 3.9.1 software (RRID:SCR_003032).

### KEGG pathways enrichment analysis and gene ontology functional analysis

Selected proteins that have interactions with gallic acid were then subjected to functional gene ontology (GO) analysis, with the purpose of being able to describe and limit gene functions that apply to all species. The method can effectively identify biological processes related to biological phenomena and is helpful for obtaining more meaningful gene functional information. GO enrichment analysis was performed with the Enrichr database ( https://maayanlab.cloud/Enrichr/). Numerous genes that are related to breast cancer and apoptosis pathways were uploaded to the database.

### Molecular modelling analysis protein selection and preparation

The macromolecules or target proteins that were used were the crystal structures of the JUN (PDB ID: 2P33), AKT1 (PDB ID:1H10), CASP3 (PDB ID:3KJF), and CASP7 (PDB ID: 5V6U) proteins which were taken from the PDB data bank ( http: //www.pdb.org/). Protein preparation was performed using AutoDock 4.2.6 software (RRID:SCR_012746). The protein was separated from the original ligand, and the water molecule was removed from the protein file, which later was ready to be saved in PBD format. Polar hydrogen atoms were added, nonpolar hydrogen atoms were removed, and a gasteiger charge was added to the protein. Binding pockets were defined by a grid map with docking grid sizes of 40x40x40, 50x50x50, and 60x60x60.

### Ligand preparation

A total of 12 gallic acid compounds and their derivatives (N-alkyl gallamide), which were later referred to as test ligands, were drawn using the Marvin Sketch software from ChemAxon (RRID:SCR_004111). ^22^ Test ligands were prepared using AutoDock 4.2.6 software by converting files in SDF format to PBDQT.

### Molecular docking

The test ligand's molecular anchorage was determined using the Lamarckian genetic algorithm ^23^ and AutoDock 4.2.6 software with the default settings. The output data was in DPF format, which was then executed via the command prompt by Autogrid4 and the Autodock4 docking process. The AutoDock 4.2.6 software was used to examine the conformation, bond affinity values, and interactions of the docking outcomes. Using Ligplot+ (RRID: SCR_018249) and LigandScout (RRID:SCR_014889) software, the visualization of protein-ligand interactions was examined.

### ADMET profiling

Analysis of the ADMET characterization profile resulting from molecular modelling was conducted using several tools. Predictive analysis of drug likeness was determined using the web-based tool SwissAdme ( http://www.swissadme.ch/). Predictive analysis of adsorption, distribution, metabolism, and excretion was performed using the pkCSM Pharmacokinetics web-based tool ( http://biosig.unimelb.edu.au/pkcsm/). Toxicity prediction analysis was conducted using the web-based tool ProTox-II (RRID:SCR_018506) Prediction ( https://tox-new.charite.de/protox_II/). Meanwhile, the value of Log D was predicted using Marvin Sketch software (Log D prediction).

## Results and discussion

Drug-protein interactions were analyzed with the lead compound, which is gallic acid. Furthermore, analysis of the interaction of gallic acid with protein was done with the STITCH network server with a high confidence level of 0.700. A total of 157 nodes (the number of interacting proteins), 1923 edges (the number of associations formed), and four interactions of gallic acid with proteins were obtained. Table 1 shows the results of the analysis of the interaction of gallic acid with proteins at a high confidence level of 0.700. Visualization of gallic acid interaction with proteins using Cytoscape software with a circular display type. ^24^

**Table 1.** The results of the analysis of the interaction of gallic acid with protein.

No.	Gallic acid interaction with:	*Combined score*
1	JUN	0.823
2	CASP3	0.745
3	AKT1	0.742
4	CASP7	0.700

Gallic acid is the leading compound in this study and was used as a drug compound to be analysed for protein-drug interactions. In addition, many studies related to gallic acid as an anticancer agent have been carried out; therefore, research data on gallic acid are widely available in the database. Due to this, it is presumed that the gallic acid derivatives (N-alkyl gallamides) have at least the same type of interaction as gallic acid. The interaction of gallic acid with breast cancer proteins and apoptosis resulted in four interactions ( table 1). ^24^ The interaction of gallic acid with four proteins, namely JUN, CASP3, AKT1, and CASP7 had a high combined score. The combined score for each protein are JUN (0,823), CASP3 (0,745), AKT1 (0,742) and CASP7 (0,700). The combined score was obtained bas on the volume of research data in the database, which includes co-expression, co-occurrence, experiment, and text mining scores. The higher the combined score of the interaction, the higher the confidence level.

JUN (c-JUN) is a transcription factor 1 (AP-1) driving protein that binds and activates transcription on the TRE/AP-1 element. Growth factors such as extra or intracellular signals, changes in onco-proteins, and UV light exposure stimulate c-JUN phosphorylation at serine 63/73 and activate c-JUN-dependent transcription. Therefore, activated c-JUN has the potential to play an essential role in carcinogenesis and cancer development. ^25^

AKT, also known as protein kinase B, is a critical element of the PI3K/AKT signalling pathway. ^26^ In addition, AKT regulates cancer characteristics such as tumor growth, as well as survival and invasion of tumor cells. AKT has three different isoforms, those being AKT1, AKT2, and AKT3. The three isoforms are reported to have specific effects on breast cancer. AKT1 plays a role in early tumors, AKT 2 is responsible for tumor development and metastasis, while AKT3 is associated with negative ER (Estrogen Receptor) status. Moreover, AKT1 increases cell proliferation through cell cycle proteins such as 21, p27, and cyclin D, and plays a role in preventing apoptosis through p53. ^27^ AKT1 was also reported to be involved in regulating breast cancer progression tested *in vivo* in ErbBB-induced mice. In this study, AKT1-deficient breast epithelial tumor cells (MEC) were reduced in size and proliferative capacity with a reduced abundance of cyclin D1 and p27. ^28^

CASP3 and CASP7 are a group of proteins involved in the process of cell apoptosis. More precisely CASP3 and CASP7 are effector caspases along with CASP6. All caspase families are inactive zymogens (pro-caspases) and their activation requires proteolytic activation during apoptosis. Effector caspases are activated by initiator caspases (CASP2, CASP8, CASP9, and CASP10) through cleavage at internal Asp residues leading to disassembly of large and small subunits, whereas inhibitory caspases are activated by dimerization via signals obtained from death receptors. ^29^ In breast cancer, CASP3 is overexpressed and is significantly associated with poor breast cancer-specific survival and provides additional prognostic value in different phenotypes. ^30^ The expression of CASP7, which is a pro-apoptotic protein sterically inhibited by XIAP protein, causes inhibited apoptosis. Higher levels of CASP7 were found in well-differentiated tumors, including ER+ breast tumors. This is due to the presence of an estrogen receptor element located in the CASP7 promoter area. CASP7 expression was significantly associated with estrogen receptor (ERα) expression status and continued to increase at the breast tumor stage. ^31^

The KEGG pathway analysis based on the interaction of gallic acid with JUN, AKT1, CASP3, and CASP7 proteins indicated several signalling pathways, including the PI3K-AKT signalling pathway, MAPK signalling pathway, estrogen signalling pathway, receptor-mediated extrinsic pathway, and TNF-signalling pathway. These signalling pathways are related to cell proliferation, the cell cycle, and apoptosis. ^26^

Selected target proteins, JUN, AKT1, CASP3, and CASP7 are predicted to play a role in the breast cancer mechanism pathway as a therapeutic pathway for gallic acid and its derivatives summarized by the KEGG database. ^26^ There are four predicted pathways, namely estrogen signalling pathways, PI3K/AKT signalling pathways, extrinsic receptor mediated pathways, and TNF-α signalling pathways. In the estrogen-mediated pathway, gallic acid and its derivatives are expected to bind to JUN proteins that can inhibit JUN activity in the cell cycle process. Whereas in PI3K/AKT signalling pathways, when AKT has been activated then gallic acid and its derivatives in the cell membrane are expected to inhibit both AKT activity and the activity of a downstream protein, namely mTOR so that the process of cancer cell proliferation can be inhibited. In contrast to JUN and AKT, whose activity is expected to be inhibited by gallic acid and its derivatives, the extrinsic and TNF-α signalling pathways are expected to activate CASP3 and CASP7 to immediately carry out the apoptotic process. According to this description, the relationship between proteins that interact with gallic acid based on protein-drug interaction analysis has a role and function in breast cancer related to the growth, development, and apoptosis of breast cancer cells.

Breast cancer protein-associated pathways and apoptosis were obtained by enrichment analysis of the KEGG pathway using the Enrichr database ( https://maayanlab.cloud/Enrichr/). One hundred and sixty-nine KEGG pathways were enriched, including breast cancer pathways and deep cancer pathways. Each enriched p value was calculated and compared with the Fisher method, where a p value<0.01 was significantly enriched. The p values were ordered from smallest to largest. The top 10 ontology gene enrichment results are presented in underlaying data. ^32^

Genes that interact with gallic acid directly or indirectly were analyzed for enrichment of the KEEG pathway in order to explore biological pathways involving related genes. The top ten KEEG pathways were significantly enriched with p values<0.001, the pathway in breast cancer was the main biological pathway enriched in related genes. Other biologic pathways in the 10 enriched pathways are associated with cancer, from colorectal cancer to endometrial cancer. The genes involved in the biological pathway of breast cancer were then analyzed for gene ontology (GO) enrichment. GO is a technique for interpreting high throughput molecular data and generating hypotheses about biological phenomena that underlies experiments or interpreting data (genes) using the ontology classification system of genealogy. ^33^

The functional enrichment analysis of GO proteins that play a role in breast cancer and the mechanism of apoptosis consists of three aspects: cell composition, biological processes, and molecular functions. The analysis was carried out through the Enrichr database ( https://maayanlab.cloud/Enrichr/). A total of 73 enrichment results we retrieved in aspects of cell composition, including the nucleus, intracellular membrane-bounded organelle, cyclin-dependent protein kinase holoenzyme complex, and other cell components. The enrichment results on aspects of biological processes showed that 1086 included negative regulation of the apoptotic process, positive gene expression regulation, fibroblast cell proliferation, and other biological processes. Meanwhile, the aspect of the molecular function illustrated 111 functions involving serine/threonine/tyrosine kinase protein activity, phosphatase binding, kinase binding, and other molecular functions. Each enriched p value was calculated and compared with the Fisher method, where p values<0.01 were significantly enriched. The p values were ordered from smallest to largest. ^34^

There are three categories in GO, namely biological processes, molecular functions, and cellular components. Biological processes refer to the biological goals to which genes or gene products contribute. The process is achieved by means of one or more ordered sequences of molecular functions. Processes often involve chemical or physical transformations. Molecular function is defined as biochemical activity including binding to specific ligands or structures of gene products. Molecular functions simply describe what is done without specifying where or when it occurs. Meanwhile, the cellular component refers to the place in the cell where the gene product is active. ^35^

In this study, the ontology gene enrichment of the genes inside the breast cancer pathway was significantly enriched with the biological process category called “negative regulation of the apoptotic process”. Negative regulation of the apoptotic process is defined as a series of gene products involved in the breast cancer pathway that inhibit apoptosis or ‘downregulate’ apoptosis. The pathway involved in this biological process is the Wnt canonical signalling pathway. Canonical Wnt signalling is the pathway that is responsible for β-catenin and T cell factor (TCF) or lymphoid enhancer factor (LEF), which is responsible for the proliferation and metastasis of breast cancer cells ^36^ and maintenance of stemness. ^37^ Wnt signaling in breast cancer is activated by loss of the TP53 gene. ^38^

Next, GO in the category of molecular function that was significantly enriched, specifically the biochemical activity of the enzyme “serine/threonine kinase”, will be discussed. Serine/threonine kinase is a member of the protein kinase superfamily that phosphorylates the amino acids serine or threonine. Through phosphorylation of protein kinases, it chemically transfers phosphate from ATP (Adenosine Triphosphate) or GTP (Guanosine Triphosphate) to targeted amino acids with the release of hydroxyl groups from their protein substrates. The phosphorylation process can induce conformational changes in the substrate protein which can disrupt protein-protein interactions. This conformational change influences its protein activity, cellular localization, or association with other proteins. These protein/enzyme kinases have been shown to regulate important molecular pathways in cellular processes including proliferation, metabolism, migration, survival, and apoptosis. ^39^ Uncontrolled kinase activity due to mutation or loss of inhibitory mediators are commonly found in cancers, including breast cancer.

In breast cancer, serine/threonine kinase activity, also known as protein kinase B/Akt, interacts with breast tumor kinase (Brk) or protein-tyrosine kinase, which is involved in growth and cell survival and is overexpressed in most breast cancers but not in normal breast epithelial cells. ^40^ Recently, one of the serine/threonine kinases (D) family, namely protein kinase D3 (PRKD3) was reported to promote the proliferation, growth, migration, and invasion of cancer cells in several types of tumors including breast cancer and it is said to be an alternative therapeutic target that is promising for cancer treatment. ^41^

The next category of GO to be discussed is the significantly enriched cellular component called “nucleus”. As previously explained, cellular components describe the sites in the cell where activities occur whereas gene products are active. In this study, many genes in the breast cancer pathway were involved in the process of cell proliferation and interact with transcription factors, where the site of the process was in the cell nucleus. In addition, the significantly enriched cellular component is “intracellular membrane-enclosed organelles” based on the definition of the ontology gene browser. These organelles are organized structures of distinctive morphology and function, bound by single or multiple lipid bilayer membranes and are found within cells. This includes the nucleus, mitochondria, plastids, vacuoles, and vesicles, but excludes plasma membranes. From this definition, there are several places in the cell that are the site of cell proliferation and apoptosis and support the objectives of this study regarding apoptotic agents.

Molecular docking analysis was used to computationally predict the interaction between proteins and the test compound (ligand). The target protein was selected based on the analysis of drug interactions with proteins with a high confidence value. These proteins include JUN (PDB ID: 2P33), AKT1 (PDB ID:1H10), CASP3 (PDB ID:3KJF), and CASP7 (PDB ID: 5V6U) taken from the PDB data bank ( http://www.pdb.org/). The protein structure that was used is a 3D crystal structure downloaded in PDB format.

AutoDock 4.2.6 software was used to prepare each protein. The water molecules were removed from the protein, and hydrogen and charge were added. The original protein and ligand were separated. The prepared proteins and ligands were subsequently stored as PDBQT files. The original protein and ligands were used to validate the grid box, which is a location for the attachment of gallic acid molecules and their derivatives. Validation of grid box dimensions began with 40x40x40, 50x50x50, and 60x60x60, or was adjusted to the original ligand size of each protein. The best validation results are chosen when the RMSD reference value is less than 2Å and the binding free energy (ΔG) is low ( table 2).

**Table 2.** Grid Box Validations.

	ΔG (kcal/mol)		
*Grid Center*	40x40x40	50x50x50	60x60x60
Protein JUN			
X = 23.679	-8.82	-9.2	-9.3
Y = 9.022			
Z = 30.409			
*RMSD references Å*	0.46	5.2	5.39
*Inhibition Constant (*nM)	340.35	181.59	151.96
Protein AKT1			
X = 15.149	-15.93	-14.72	-14.76
Y = 24.335			
Z = 16.343			
*RMSD references Å*	1.46	2.1	1.35
*Inhibition Constant* (pM)	2.12	16.31	15.26
Protein CASP3			
X = -44.236	-8.47	-8.1	-8.97
Y = 9.243			
Z = -21.625			
*RMSD references Å*	1.63	3.88	1.51
*Inhibition Constant* (nM)	613.63	1.15 (μM)	265.29
Protein CASP7			
X = -42.505	-7.41	-7.55	-7.15
Y = 12.409			
Z = 10.242			
*RMSD references Å*	2.03	1.27	1.82
*Inhibition Constant* (μM)	3.7	2.9	5.76

Gallic acid and its derivatives, along with tamoxifen used as a control, were then used as test ligands for molecular modeling these proteins with the best grid box. The results of molecular docking are predictive values of binding free energy, inhibition constants, and bond interactions between test ligands and protein amino acid residues. The results of the molecular docking of 13 test compounds to four proteins are shown in table 3.

**Table 3.** The results of the molecular docking analysis of gallic acid and N-alkyl gallamide.

No	Compounds	JUN		AKT1	
		ΔG (kcal/mol)	Ki (μM)	ΔG (kcal/mol)	Ki (μM)
1.	Gallic acid	-4.82	292.07	-7.80	1.93
2.	Tamoxifen	-7.42	3.65	-4.38	611.97
3.	N-Methyl Gallamide	-5.53	87.83	-5.86	50.89
4.	N-Ethyl Gallamide	-5.86	50.53	-5.99	40.80
5.	N-Propyl Gallamide	-6.16	30.47	-6.06	36.07
6.	N-Butyl Gallamide	-6.31	23.77	-5.96	42.60
7.	N-Sec-Butyl Gallamide	-6.40	20.43	-6.20	28.52
8.	N-Ters-Butyl Gallamide	-6.57	15.41	-6.51	17.02
9.	N-Amyl Gallamide	-6.51	16.78	-5.88	48.82
10.	N-Isoamyl Gallamide	-6.74	11.52	-6.23	27.34
11.	N-Hexyl Gallamide	-6.73	11.68	-5.82	54.28
12.	N-Heptyl Gallamide	-6.73	11.69	-5.39	111.51
13.	N-Octyl Gallamide	-6.99	7.46	-5.27	138.07

No	Compounds	CASP3		CASP7	
		ΔG (kcal/mol)	Ki (μM)	ΔG (kcal/mol)	Ki (μM)
1.	Gallic acid	-5.17	161.71	-6.45	18.78
2.	Tamoxifen	-6.34	22.63	-8.13	1.09
3.	N-Methyl Gallamide	-5.78	57.63	-6.24	26.64
4.	N-Ethyl Gallamide	-5.93	44.74	-6.40	20.45
5.	N-Propyl Gallamide	-5.81	54.73	-6.58	15.09
6.	N-Butyl Gallamide	-5.92	45.61	-6.87	9.16
7.	N-Sec-Butyl Gallamide	-6.15	30.92	-6.89	8.83
8.	N-Ters-Butyl Gallamide	-6.57	15.39	-6.67	12.95
9.	N-Amyl Gallamide	-6.20	28.37	-7.07	6.62
10.	N-Isoamyl Gallamide	-6.19	28.86	-7.27	4.72
11.	N-Hexyl Gallamide	-6.30	24.05	-7.09	6.34
12.	N-Heptyl Gallamide	-5.79	57.00	-6.66	13.19
13.	N-Octyl Gallamide	-6.05	36.47	-7.08	6.48

Next, the best five ranked compounds were selected based on the results of molecular anchoring with the smallest G and Ki values for each protein. A total of nine gallic acid derivatives were obtained which were candidates for further analysis, namely ADMET analysis. ADMET analysis uses the SwissAdme webserver, pkCSM, Marvin Sketch, and the ProtoxII Prediction webserver. Table 4 shows the predicted results of ADMET analysis of gallic acid and the nine gallic acid derivatives N-alkyl gallamide.

**Table 4.** ADMET analysis prediction of gallic acid and their derivatives (N-Alkyl gallamide).

Compound	Drug Likeness Lipinski					Adsorption		Distribution
	BM	HBA	HBD	Log P	TPSA	Gi Absorption	P-gp Substrate	Fraksi un-bound
(1)	170.12	11	4	0.50	97.99	43.37	No	0.617
(2)	197.19	4	4	0.58	89.79	72.86	yes	0.642
(3)	211.21	4	4	0.90	89.79	71.29	yes	0.611
(4)	281.35	4	4	2.64	89.79	90.44	yes	0.442
(5)	253.29	4	4	1.90	89.79	91.15	yes	0.515
(6)	267.32	4	4	2.23	89.79	90.79	yes	0.479
(7)	239.27	4	4	1.57	89.79	91.75	yes	0.550
(8)	197.19	4	4	0.58	89.79	90.71	yes	0.548
(9)	211.21	4	4	0.87	89.79	94.18	yes	0.699
(10)	225.24	4	4	1.13	89.79	93.75	yes	0.558

Compound	Distribution	Metabolism				Excretion	Toxicity	
		Inhibitor						
	BBB	CYP 2C19	CYP 2C9	CYP 2D6	CYP 3A4	Total Clearances	LD50	AOT
(1)	-1.102	No	No	No	No	0.518	2000	IV
(2)	-0.982	No	No	No	No	0.624	2000	IV
(3)	-1.016	No	No	No	No	0.310	2000	IV
(4)	-1.156	No	No	yes	No	0.978	2000	IV
(5)	-1.097	No	No	No	No	0.356	2000	IV
(6)	-1.125	No	No	No	No	0.373	2000	IV
(7)	-1.071	No	No	No	No	0.339	2000	IV
(8)	-0.794	No	No	No	No	0.216	2000	IV
(9)	-1.101	No	No	No	No	0.261	2000	IV
(10)	-0.0996	No	No	No	No	0.499	2000	IV

**Description**: **Compound** (1): Gallic acid, (2): N-Ethyl gallamide, (3): N-Profile gallamide, (4): N-Octyl gallamide, (5): N-Hexyl gallamide, (6): N-Heptyl gallamide, (7): N-Amyl gallamide, (8): N-Isoamil gallamide, (9): N-Sec Butyl gallamide, (10): N-Ters-butyl gallamide. **P-gp Substrate**: yes, indicates the compound is predicted to be P-gp substrate, no indicates the compound is not predicted to be P-gp Substrate. **CYP inhibitors**: yes, it shows that the compound can inhibit CYP inhibitors, but it doesn't show that the compound does not inhibit CYP inhibitors. **BM**: Molecular Weight, **HBA**: Hydrogen Bond Acceptor, **HBD**: Hydrogen Bond Donor, **TPSA:** Total Polar Surface Area, **Log P**: Octanol/water solubility **, Gi Absorption**: Gastro Intestinal Absorption, **Un-bound fraction**: unbound fraction of drug molecules. **BBB**: Blood Brain Barrier, **LD50**: Lethal Dose 50%, **AOT**: Acute of Toxicity.

Based on the log P characteristic profile which interprets the solubility of the octanol/water phase and the permeability of gallic acid compounds and their derivatives, it can be classified in the biopharmaceutical classification system (BCS) by following the classification system: class I (high solubility and high permeability), class II (low solubility and high permeability), class III (high solubility and low permeability), and class IV (low solubility and low permeability). Predictions of the biopharmaceutical classification system for gallic acid compounds and their derivatives (N-alkyl gallamides) are summarized in table 5.

**Table 5.** Prediction of the Biopharmaceutical Classification System (BCS) for gallic acid compounds and their derivatives (N-Alkyl Gallamide).

Compound	Solubility		Permeability		BCS Class
	Log p	Level	Gi Absorption	Level	
Gallic Acid	0.50	High	43.37	High	I
N-Ethyl Gallamida	0.58	High	72.86	High	I
N-Propyl Galamida	0.90	High	71.29	High	I
N-Octyl Gallamida	2.64	High	90.44	High	I
N-Hexyl Gallamida	1.90	High	91.15	High	I
N-Heptyl Gallamida	2.23	High	90.79	High	I
N-Amyl-Gallamida	1.57	High	91.75	High	I
N-Isoamyl Gallamida	0.58	High	90.71	High	I
N-Sec Butyl Gallamida	0.87	High	94.18	High	I
N-Tert Butyl Gallamida	1.13	High	93.75	High	I

**Description:** Log P: Octanol/water Solubility, Gi Absorption: Gastro Intestinal Absorption

In addition, the relationship between pH and Log D, which shows the distribution coefficient of gallic acid compounds and their derivatives, is determined. The purpose of this predictive analysis is to measure the lipophilicity of ionizable compounds, where the partition is a function of pH. Table 6 shows the predicted relationship between pH values and Log D of gallic acid compounds and their derivatives (N-alkyl gallamides).

**Table 6.** Prediction of the relationship between pH and Log D of gallic acid compounds and their derivatives (N-Alkyl gallamide).

pH	Log D									
	(1)	(2)	(3)	(4)	(5)	(6)	(7)	(8)	(9)	(10)
1.5	0.71	0.43	0.90	2.88	2.09	2.49	1.69	1.63	1.31	0.92
5	-0.38	0.43	0.90	2.88	2.09	2.49	1.69	1.63	1.31	0.92
6.5	-1.82	0.42	0.89	2.87	2.08	2.47	1.68	1.62	1.30	0.91
7.4	-2.55	0.34	0.81	2.79	2.00	2.40	1.60	1.54	1.22	0.83

**Description**: Compound (1): Gallic acid, (2): N-Ethyl gallamide, (3): N-Profile gallamide, (4): N-Octyl gallamide, (5): N-Hexyl gallamide, (6): N-Heptyl gallamide, (7): N-Amyl gallamide, (8): N-Isoamyl gallamide, (9): N-Sec Butyl gallamide, (10): N-Ters-butyl gallamide

The selection of the five best ranked gallic acid derivative compounds was made based on its binding values of ΔG and Ki (ordered from highest affinity). Ligand and amino acid interactions were visualized using LigPlot+ and LiganScout software. The interactions formed between the ligands and the amino acid residues of each protein are in the form of hydrophobic interactions and hydrogen bond interactions. Each interaction formed on the test ligand was compared with the original ligand. An example of visualization of the interaction of amino acid residues with ligands using LigPlot+ and LigandScout can be seen in underlaying data. ^42^ Hydrophobic interactions are shown in red semicircles, while hydrogen bond interactions are denoted by green dashed lines. The numbers on the hydrogen bond indicates the hydrogen bond distance between the amino acid residue and the ligand in angstrom units (Å).

The absorption of drug compounds and evaluation of drug likeness properties can be predicted by Lipinski's five of rules. These rules describe the molecular properties which are important for the pharmacokinetics of drug candidates. The Lipinski’s data of the drug candidate provides information about its similarity as a ligand of the drug. A good drug candidate will have Lipinski's rule of five data as follows, it has a molecular weight less than 500 Da, it does not have a hydrogen bond acceptor (HBA) of more than 10, it does not have a hydrogen donor bond (HBD) of more than 5, it has a log P value of no more than 5, and it must have a total polar surface area (TPSA) less than 140. ^43^ A complete prediction of the ADMET characteristic profile is shown in table 4.

ADMET is used to describe the absorption, distribution, metabolism, excretion, and toxicity of drugs. *In-silico* ADMET profiling is a useful tool for predicting the pharmacological and toxicological properties of drug candidates, especially at the pre-clinical stage. To improve the prediction of ADMET, an *in-silico* model has been applied. The use of these models in particular has contributed to drug optimization and the avoidance of late-stage failures, which is also important because such failures lead to unproductive investments of time and money.

The absorption is predicted based on the nature of solubility in water (Log P), lipophilicity, and absorption in the human intestinal tract (Gi absorption). Based on table 1, the predicted solubility of Log P for all gallic acid compounds and their derivatives (N-alkyl gallamide) is less than 3. This shows that these compounds have good solubility. Log P also describes the role of compound hydration, the higher the log P value, the worse the hydration. The limit of a good log P value ranges from 2 to 3. ^44^ Predicted absorption in the human intestine of gallic acid compounds and their derivatives is high because they can absorb more than 30% of the compound. Gi absorption is also associated with the permeability of drug compounds in the body.

Distribution was predicted using P-glycoprotein (P-gp) substrate, blood-brain barrier (BBB) permeability, and non-segmentation-related descriptors. The descriptors were predicted using pkCSM-pharmacokinetics. P-gp is an ATP-dependent drug extraction pump and is found in various human tissues. All newly synthesized molecules must be P-gp substrates. According to the predicted results in this study, only gallic acid compounds were not predicted as p-gp substrates. The BBB is a complex structure that separates the central nervous system (CNS) from peripheral tissues. To maintain homeostasis in the CNS, the BBB controls the transfer of matter, nutrients, and cells from the blood to the brain and from the brain to the blood. It also participates in the clearance of cellular metabolites and toxins from the brain to the blood. ^44^ The BBB permeability values of our compounds were estimated to range from −0.0996 to −1.156.

The drug molecules in the plasma are in balance between being bound to serum proteins and unbound. The proportion of drug molecules in plasma that are not bound to proteins is called the “un-bound fraction”. This profile influences renal glomerular filtration and hepatic metabolism, resulting in values for the volume of distribution, total clearance, and drug efficacy. ^45^ The higher the binding of the drug to proteins in the blood, the less efficient it is to spread across cell membranes. In this study, the predictive value of the unbound fraction was large (from a scale of 0 to 1), which means that the binding of the drug to the protein in the blood is not too strong so that it can spread throughout the cell membrane well.

To make a candidate drug compound, it is also necessary to analyze the pharmacokinetics of the compound against cytochrome P450 (CYP) protein inhibitors. These proteins are marker proteins to determine the effect of the response of an anticancer drug and are metabolic enzymes in the liver. The human CYP enzymes important in drug metabolism are CYP 1A2, the CYP 2C family, CYP 2D6, and CYP 3A4. Therefore, in this study, we investigated whether gallic acid derivative compounds did not have an inhibitory effect on these enzymes. Based on table 1 only N-octyl gallamide compounds had an inhibitory effect on the CYP2D6 enzyme.

Excretion occurs primarily as a combination of hepatic and renal clearance, is associated with bioavailability, and is important for determining dose levels to achieve steady-state concentrations. The predicted excretion values, using the pkCSM-pharmacokinetic total clearance descriptor, ranged from 0.216 to 0.978 ml/min/kg.

In addition, the toxicity of the drug compound was also analyzed to determine whether the drug compound is toxic or not, which aims to predict the level of safety for its use. The predicted toxicity profile in this study is AOT (acute of toxicity) which is related to LD50. AOT has several classes, namely class 1 (fatal if swallowed, LD50=5 mg/kg), class II (fatal if swallowed, 5<LD50 50), class III (toxic if swallowed, 50<LD50 300), class IV (harmful if swallowed, 300<LD50 2000), class V (possibly harmful if swallowed, 2000<LD50 5000), and class V1 (non-toxic, LD50>5000). ^46^ Based on this category gallic acid and its derivatives fall into class IV.

It has been discussed previously that the solubility and permeability profile of gallic acid and its derivatives (N-alkyl gallamide) are important in predicting the pharmacokinetic properties of drug candidates. Based on this, this study tries to classify these profiles into the biopharmaceutical classification system (BCS). Based on this classification, gallic acid and its derivatives are classified in class 1 BCS, because all compounds have high solubility and permeability ( table 5).

Another important profile is the relationship between pH and Log D of gallic acid compounds and their derivatives. Log D is the distribution coefficient described for ionizable compounds because log D is a measure of the pH-dependent difference in solubility of all species in the octanol/water system. The importance of predicting the value of log D is to predict the permeability of drug compounds *in vivo.* The relationship with pH indicates that changes in the pH environment that may occur in orally administered compounds occur in the gastrointestinal tract. ^47^ This is because there is no constant pH in the body so pH is highly considered when predicting *in vivo* behavior of drug candidates. The pH range that is the primary focus of attention is pH 7.4, which is the physiological pH of blood serum, while the pH of the stomach is pH 1–3 and the ileum is pH 7–8. In this study, predictions of pH and log D values were made for gallic acid and its derivatives ( table 6). Based on the prediction results, both gallic acid compounds and their derivatives have varying Log D values. Drug candidate compounds that have a high Log D value are highly lipophilic (Log D at pH 7.4>3.5) tend to have poor water solubility which can interfere with intestinal absorption. Based on this, the log D value of gallic acid compounds and their derivatives can be concluded to have good distribution coefficients as drug candidates

The determination of the five best gallic acid derivatives based on ADMET analysis could not be determined because all test parameters were met. The selection of five gallic acid derivatives was determined by ordering the binding energy values of G and Ki as a result of molecular docking analysis ( table 3). Hydrogen bonding and hydrophobic bonding interaction between gallic acid derivative and amino acid residues in JUN, AKT1, CASP3, CASP7 proteins is presented in table 7 and visualized in underlaying data bellow. The number of hydrogen and hydrophobic bonds is compared with the original ligand interactions. In JUN proteins, in general, there are two hydrogen bonds in the amino acids Met149 and Asn152, this is different from the N-octyl gallamide compound which only has hydrogen bonds in Met149. However, the hydrophobic interactions of the N-octyl gallamide compound with JUN were more than other compounds. In addition, the JUN showed interaction with essential amino acid residues of gallic acid derivative compounds, namely Met149, Leu148, Lys93, Met146, Leu206, Val196, Ile70, and Val78.

**Table 7.** Analysis of the interaction of ligands with amino acid residues JUN, AKT1, CASP3 and CASP7.

a. Ligand interaction with amino acid residue of JUN						
Amino acid	Native ligand	(2)	(7)	(4)	(5)	(9)
Met49	√ (H: 2,77; 2,86)	√ (H: 3,02)	√ (H: 2,90)	√ (H: 3,06)	√ (H: 2,86)	√ (H: 3,19)
Leu148	√	√	√	√	√	√
Ala91	√	√	√	√	√	√
Glu147	√	√	√	√	√	√
Ile124	√	√	√	√	√	√
Lys93	X	√	x	X	√	X
Met146	√	√	√	√	√	√
Leu206	√	√	√	√	√	√
Val196	√	√	√	√	√	√
Asn152	√ (H: 2,85)	√	√ (H: 3,18)	√ (H: 3,17)	√ (H: 3,17)	√ (H: 3,06; 3,09)
Ala151	√	√	√	√	√	√
Asp150	√	√	√	√	√	√
Ile70	√	√	x	X	x	X
Asp207	X	√	x	X	x	X
Glu111	X	√	x	x	x	X
Val78	√	x	√	x	x	√
Gln155	√	x	x	x	x	X
Gly71	√	x	x	x	x	X
Ser72	√	x	x	x	x	X

b. Ligand interaction with amino acid residue of AKT1						
Amino Acid	Native Ligand	(9)	(7)	(8)	(2)	(1)
Glu17	√ (H: 2,83;3,16)	√ (H: 2,92)	√ (H: 2,95)	√ (H: 2,78)	√ (H: 2,87)	√ (H: 2,89)
Gly16	√	√	√	√	√	√
Lys14	√ (H: 2,64)	√	√	√	√	√
Arg25	√ (H: 2,91;3,33)	√ (H: 2,83)	√ (H: 2,88)	√ (H: 2,89)	√ (H: 2,84)	√ (H: 2,88)
Asn53	√ (H: 2,63;3,02)	√ (H: 3,06)	√ (H: 3,04)	√ (H: 3,08)	√ (H: 3,05)	√ (H: 3,06)
Arg23	√ (H: 2,76;3,08)	√	√ (H: 2,96;3,18)	√ (H: 3,00;3,34)	√ (H: 3,08)	√ (H: 3,05)
Ile19	√ (H: 2,95)	√	√	√	√	√
Tyr18	√	√	√	√	√	√
Arg86	√ (H: 2,89;2,93)	x	x	x	x	x
Phe55	√	x	x	x	x	x

c. Ligand interaction with amino acid residue of CASP3						
Amino Acid	Native Ligand	(9)	(4)	(6)	(7)	(8)
Trp206	√	√	√	√	√	√
Phe250	√	√	√	√	√	√
Ser249	√	√	√	√	√	√
Glu248	√	√ (H: 3,22)	√	√	√	√
Trp214	√	√	√	√	√ (H: 2,75)	√
Phe247	x	√	√	√	√	√
Asn208	x	√ (H: 3,03)	√ (H: 2,99)	√ (H: 2,96)	√	√ (H: 2,81)
Arg207	√ (H: 2,64; 2,77;2,90; 3,01)	√ (H: 2,72)	√	√	√ (H: 2,79)	√
Ser251	x	x	√	√	√	X
Phe256	√	x	√	√	x	X
Glu246	x	x	x	x	√	X
Tyr204	√	x	x	x	x	X
Ser205	√ (H: 2,62)	x	x	x	x	X

d. Ligand interaction with amino acid residue of CASP7						
Amino Acid	Native Ligand	(7)	(4)	(3)	(6)	(8)
Arg167	√ (H:2,90)	√	√	√	√	√
Phe219	√	√	√	√	√	√
Glu216	x	√	√	√	√	√
Lys160	√ (H:2,79)	√	√	√	√	√
Ile159	√	√	√	√	√	√
Asn148	x	√	√	√	√	x
Tyr223	√	√	√	√	√	√
Phe221	√	√	√	√	√	√
Thr163	√	√ (H: 2,94)	√ (H: 3,07)	√ (H: 3,13)	√ (H: 2,99)	√ (H: 2,75)
Ala164	√	√	√	√	√	√
Pro158	x	x	x	√	x	x
Met294	√	x	x	√	x	x

**Description**: (1) N-Ethyl gallamide, (2) N-Propyl gallamide, (3) N-octyl gallamide, (4) N-Hexyl gallamide, (5) N-Hepthyl gallamide, (6) N-Amyl gallamide, (7) N-Isoamyl gallamide, (8) N-Sec-Butyl gallamide, (9) N-Ters-Butyl gallamide. (√) no interaction, (X) no interaction.

In the AKT1 protein, the hydrogen bonding interactions on the original ligand were more than in the test compound. There were seven hydrogen bonds in the original ligand and three hydrophobic interactions, while the N-ters-butyl gallamide compound has three hydrogen bonds (Glu17, Arg25, Asn53) and five hydrophobic interactions. While the other four compounds have four hydrogen bonds and hydrophobic bonds, with hydrogen bonds at the same amino acid residues (Glu17, Arg25, Asn53, Arg23). Of all the interacting amino acids, there were only two essential amino acids that interacted with the test compound on the AKT1 protein, namely Lys14 and Ile19.

In CASP3 protein, the N-ters-butyl gallamide compound had more hydrogen bonds with amino acids than the original ligand and four other compounds. Amino acids that have hydrogen bonding interactions in N-ters-butyl gallamide compounds are Glu248, Asn208, and Arg207. Meanwhile, the essential amino acids that interact with the test compound on CASP3 protein are Trp206, Trp214, Phe250, Phe247, and Phe256. The essential amino acid residue that is hydrogen bonded with the N-isoamyl gallamide compound is Trp214.

In CASP7 protein, hydrogen bond interactions in the native ligand were more than that of the test compound. But uniquely, hydrogen bonds are formed on the same amino acid residues in all test compounds, namely Thr163, which is one of the essential amino acids. The hydrophobic bond interactions formed were also generally the same for all test compounds. The interacting essential amino acids include Phe219, Lys160, Ile159, Phe221, Thr163, and Met294.

Based on the study of the interaction of the test compounds with each target protein, the types of interactions formed are mostly similar to the original ligands for each protein. It can be stated that the test compound is attached to each protein in the same coordinates as the original ligand. In addition, the test compound for gallic acid and its derivatives is expected to have the same activity as the original ligand on each target protein.

*In-silico*, the selection of the best compounds derived from gallic acid as candidates for apoptotic agents involves several stages of selection. First, the designed gallic acid derivatives were subjected to molecular docking analysis with the selection criteria being the order of low bond energy (ΔG). The lowest (most negative) binding energies were ranked and the best 5 compounds were selected from each target protein. Second, the five best gallic acid derivatives based on the results of molecular docking analysis were selected based on the characteristic profile of ADMET, with the selection criteria being compounds that have a good ADMET characteristic profile and are non-toxic. However, in this study, the selection based on the characteristic profile of ADMET could not be determined, because gallic acid derivative compounds have good ADMET profiles as drug candidates.

Based on the results of molecular docking and ADMET profiling, the best of three gallic acid derivatives, namely N-octyl gallamide, N-ters-Butyl gallamide and N-isoamyl gallamide have a potency to be further developed as a promising anti-breast cancer agent.

## Conclusion

There are several protein-protein interactions involved in the incidence of breast cancer and apoptosis against gallic acid compounds with a high level of confidence, namely JUN, AKT1, CASP3, and CASP7. Gallic acid derivative compounds (N-alkyl gallamide) can be characterized based on ADMET properties, and molecular docking predictions can be determined. The best gallic acid derivative compounds based on this study are N-Octyl gallamide, N-Ters-butyl gallamide, and N-Isoamyl gallamide.

## Data Availability

Figshare: 2D chemicals structure of gallic acid and it derivatives (N-Alkyl gallamide). https://doi.org/10.6084/m9.figshare.21391545.v1. ^22^ Figshare: Interaction of gallic acid with protein. https://doi.org/10.6084/m9.figshare.21391902.v1. ^24^ Figshare: Systematic prediction of four target proteins and gallic acid therapeutic pathways. https://doi.org/10.6084/m9.figshare.21391524.v1. ^26^ Figshare: Top 10 KEGG pathway enrichment analysis results. https://doi.org/10.6084/m9.figshare.21392082.v1. ^32^ Figshare: Top 10 ontology gene enrichment analysis results. https://doi.org/10.6084/m9.figshare.21392232.v1. ^34^ Figshare: Visualization of Ligand Interactions with Protein Amino Acid Residues. https://doi.org/10.6084/m9.figshare.21392520.v1. ^42^ Biostudies: Raw data gene list from KEGG with accession number S- BSST934 ^21^ Data are available under the terms of the Creative Commons Zero “No rights reserved” data waiver (CC0 1.0 Public domain dedication).
