# Molecular docking, ADMET profiling of gallic acid and its derivatives (N-alkyl gallamide) as apoptosis agent of breast cancer MCF-7 Cells

**DOI:** 10.12688/f1000research.127347.3

**Published:** 2024-02-08

**Authors:** Ade Arsianti, Norma Nur Azizah, Linda Erlina

**Affiliations:** 1 Master’s Programme in Biomedical Science, Faculty of Medicine, Universitas Indonesia, Jakarta, 10360, Indonesia; 2 Drug Development Research Center, Indonesian Medical Education and Research Institute (IMERI), Jakarta, 10630, Indonesia; 3 Department of Medical Chemistry, Faculty of Medicine, Universitas Indonesia, Jakarta, 10360, Indonesia; 4 Bioinformatics Core Facilities, Indonesian Medical Education and Research Institute (IMERI), Jakarta, 10630, Indonesia

**Keywords:** N-Alkyl gallamide, molecular docking, ADMET, breast cancer

## Abstract

**Background:**

In 2020, breast cancer has become the most common cancer in the world and in Indonesia. Searching for anticancer drugs using computational methods is considered more effective and selective than other methods. Gallic acid and its derivatives (esters and amides) are compounds that have biological activities such as anticancer effects. The purpose of this study was to analyse the molecular modelling and ADMET (Adsorption, Distribution, Metabolism, Excretion and Toxicity) profile of gallic acid derivative compounds (N-alkyl gallamides) as anticancer agents.

**Methods:**

Target proteins were selected by analysis of protein-protein and drug-protein interactions. Molecular modelling was done by molecular docking and molecular dynamic simulation. Predictive analysis of the ADMET profile of gallic acid and its derivatives (N-alkyl gallamide) was conducted using Marvin Sketch, Swissadme, protox II, and pkCSM pharmacokinetics. The selected target proteins were JUN, AKT1, CASP3, and CASP7.

**Results:**

Compounds N-octyl gallamide, N-ters-butyl gallamide, and N-isoamil gallamide were the three best gallic acid derivatives based on molecular modelling analysis of target proteins associated with breast cancer. The ADMET profile of the N-alkyl gallamide compound is predictable and shows a good profile as a candidate for anticancer drugs.

**Conclusion:**

N-octyl gallamide, N-ters-butyl gallamide, and N-isoamil gallamide have potential as anti-breast cancer agents.

## Introduction

Cancer is ranked second globally in diseases that could lead to death and caused 10 million deaths in 2020. Generally, the most prevalent types of cancer are breast, colorectal, and lung cancers. ^1^^,^^2^ Based on Global Cancer Observatory (GLOBOCAN), cancer cases in Indonesia increased in 2020, reaching 396,914 cases with a total death toll of 234,511. The leading forms of cancer were breast and cervical cancers, with the number of cases being 65,858 (16,6%) and 36,633 (9,2%), respectively. ^3^

Over recent years, research on anticancer therapies has been increasing, using various materials as anticancer drugs, which could be obtained via synthetic methods. ^4^^–^^6^ Although many potential anticancer drugs have been synthesized, the medical needs are far from fulfilled. Various factors could affect the insufficiency of these drugs to meet these needs, including selectivity limitations of conventional drugs that could lead to toxicity, as well as the presence of metastasis and multi-drug resistance. ^7^^,^^8^ The search for selective and effective anticancer agents can be improved by computational methods. Computational methods have been used to significantly accelerate the drug design process. Protein-protein interactions, drug-protein interactions, and molecular modeling are the easiest methods to find and predict new molecules as potential drugs with shorter time and lower costs than conventional methods. ^9^

Computer-based molecular modelling merges informatics methods, medical sciences, and biophysics. The combination of these fields could predict the efficiency of potential therapeutic molecules that are designed before *in vitro* and pre-clinical tests. ^10^ Characteristics and profiles of adsorption, distribution, metabolism, excretion, and toxicity (ADMET), become one of the important parameters in the discovery and development of drug candidates to determine the feasibility of drug candidates before being developed in the next stage. ^10^

In recent decades, gallic acid has become a compound that is in great demand as an anticancer therapeutic agent. Gallic acid (3,4,5-trihydroxybenzoic acid) is widely contained in natural ingredients which has been reported to have antioxidant, antifungal, antiviral, anti-inflammatory, and anticancer activities. ^11^ Gallic acid has also been reported to have biological activity against several cancer cell lines such as leukemia, lung cancer, colorectal cancer, and breast cancer cell lines and does not show biological activity against normal lymphocyte cells. ^12^^,^^13^

According to Silva *et al*. (2017), gallic acid and its derivatives (alkyl esters) have pro-apoptosis activity and show as non-genotoxic and non-mutagenic agents to prevent chromosomal damage due to chemical exposure. ^14^ However, because gallic acid is a hydrophilic compound, it is more difficult for it to penetrate the hydrophobic cell membrane. Dorothea *et al*., (2016) concluded that increasing the potential for cytotoxicity to MCF7 breast cancer cells, lipophilicity must be achieved by modifying the structure of gallic acid by adding a lipophilic group to make it more hydrophobic. ^15^ According to in silico study, gallic acid and its derivatives (alkyl ester) can be BRAF inhibitor of colorectal cancer, ^16^ as well as inhibitor of the anti-apoptosis protein Bcl-xL in breast cancer. ^17^^,^^18^ In addition, gallic acid derivative compounds (N-alkyl gallamide) have strong cytotoxic activity on MCF7 breast cancer cells, and colorectal cancer cells, HCT 116. ^19^^,^^20^ This study aims to design gallic acid derivative compounds (N-alkyl Gallamide), analyze molecular modeling of target proteins associated with breast cancer, and determine the ADMET profile of these compounds as apoptosis agents of MCF-7 breast cancer cells.

## Methods

### Protein data collection

Protein or gene data that are related to the incidence of breast cancer were downloaded from the KEGG (Kyoto Encyclopedia of Genes and Genome) database with keywords human diseases and breast cancer. In addition, apoptosis-related genes were searched and downloaded. The list of gene names was downloaded by copying all the genes into a Microsoft Excel (RRID:SCR_016137) sheet. The list of gene names is shown in repository data with accession number S-BSST934. ^21^

### Drug-protein interactions

The list of genes or proteins that were downloaded were then analyzed for interactions between gallic acid as a drug and a list of genes associated with breast cancer and apoptosis as target proteins. This analysis used a website-based tool called STITCH ( http://stitch.embl.de/). The list of genes and gallic acid was uploaded to the STITCH webserver with the search multiple names parameter, and the selected organism as *Homo sapiens.* The confidence score was also regulated in this analysis. The selected confidence score was the high confidence category (0.700). The interaction results were later downloaded in the tab separated values (TSV) format. Visualization of drug-protein interactions was performed using Cytoscape 3.9.1 software (RRID:SCR_003032).

### KEGG pathways enrichment analysis and gene ontology functional analysis

Selected proteins that have interactions with gallic acid were then subjected to functional gene ontology (GO) analysis, with the purpose of being able to describe and limit gene functions that apply to all species. The method can effectively identify biological processes related to biological phenomena and helps obtain more meaningful gene functional information. GO enrichment analysis was performed with the Enrichr database ( https://maayanlab.cloud/Enrichr/). Numerous genes that are related to breast cancer and apoptosis pathways were uploaded to the database.

### Molecular modeling analysis protein selection and preparation

The macromolecules or target proteins that were used were the crystal structures of the JUN (PDB ID: 2P33), AKT1 (PDB ID:1H10), CASP3 (PDB ID:3KJF), and CASP7 (PDB ID: 5V6U) proteins which were taken from the protein data bank (PDB). Protein data bank can be accessed through the website http: //www.pdb.org/. Protein preparation was performed using AutoDock 4.2.6 software (RRID:SCR_012746). The protein was separated from the original ligand, and the water molecule was removed from the protein file, which later was ready to be saved in PBD format. Polar hydrogen atoms were added, nonpolar hydrogen atoms were removed, and a gasteiger charge was added to the protein. Binding pockets were defined by a grid map with docking grid sizes of 40x40x40, 50x50x50, and 60x60x60.

### Ligand preparation

A total of 12 gallic acid compounds and their derivatives (N-alkyl gallamide), which were later referred to as test ligands, were drawn using the Marvin Sketch software from ChemAxon (RRID:SCR_004111). ^22^ Test ligands were prepared using AutoDock 4.2.6 software by converting files in structure data file (SDF) format to protein data bank, partial charge (Q), and atom type (T) (PBDQT) format.

### Molecular docking

The test ligand's molecular anchorage was determined using the Lamarckian genetic algorithm ^23^ and AutoDock 4.2.6 software with the default settings. The output data was in dynamic process format (DPF), which was then executed via the command prompt by Autogrid4 and the Autodock4 docking process. The AutoDock 4.2.6 software was used to examine the conformation, bond affinity values, and interactions of the docking outcomes. Using Ligplot+ (RRID: SCR_018249) and LigandScout (RRID:SCR_014889) software, the visualization of protein-ligand interactions was examined.

### Adsorption, Distribution, Metabolism, Excretion and Toxicity (ADMET) Profiling

Analysis of the ADMET characterization profile resulting from molecular modelling was conducted using several tools. Predictive analysis of drug likeness was determined using the web-based tool SwissAdme ( http://www.swissadme.ch/). Predictive analysis of adsorption, distribution, metabolism, and excretion was performed using the pkCSM Pharmacokinetics web-based tool ( http://biosig.unimelb.edu.au/pkcsm/). Toxicity prediction analysis was conducted using the web-based tool ProTox-II (RRID:SCR_018506) Prediction ( https://tox-new.charite.de/protox_II/). Meanwhile, the value of Log D was predicted using Marvin Sketch software (Log D prediction).

### Cytotoxic activities using MTT assay

A total of 10,000 cells were placed on 96-well plates per well. Cells were incubated in a 5% CO _2_ incubator for 24 hours. After 24 hours, the cells were observed. If the cells have grown on the bottom of the well plate, then the sample (N-Alkyl Gallamide) can be added with different concentration variations, namely 200 μg/mL, 100 μg/mL, 50 μg/mL, 25 μg/mL, 12.5 μg/mL, 6.25 μg/mL, and 3.125 μg/mL dissolved in complete medium. After that, the sampled cells were incubated again for 24 hours.

The cells were observed, the samples were discarded, and the cells were added to the MTT reagent at a concentration of 5 mg/mL with a dilution of 10 times. Cells were given MTT containing as much as 100 μl and then incubated for 3–4 hours. The formazan crystals formed were dissolved by adding DMSO (Dimethyl Sulfoxide) and then read using an ELISA reader with a wavelength of 590 nm. The absorbance obtained is used to make a relationship curve between concentration and inhibition (% inhibition). The inhibition concentration 50 (IC50) value can be determined from this curve.

### Apoptosis test against MCF7 cells using flow cytometry

The IC _50_ value is used as the basis for the concentration of the compound to be tested for its apoptosis activity by the flow cytometry method. This analysis method uses the Annexin V-FITC Apoptosis Detection Kit from SIGMA. A total of 100,000 cells were grown, incubated for 24 hours in a 5% CO _2_ incubator, and treated with N-Alkyl gallamide at the same concentration as IC _50_ for 24 hours. The treated cells were harvested and washed using cold PBS (Phosphate Buffer Saline). Then the washed cells were homogenized using one binding buffer with a total volume of 500 μL. The cells treated with binding buffer were then divided into 2 tubes, each containing 250 μL of cells. The first tube is a tube without the addition of dye (unstained), while the second tube (stain) has as much as 5 μL of Annexin V-FITC and 5 μL of propidium iodide (PI). The mixture (Annexin and PI) was incubated for 15 minutes in the dark room and then quantified by BD FACS Aria III flow cytometry.

### Molecular dynamic simulation

The molecular dynamics simulation of the N-Alkyl Galamide-CASP3 complex resulting from docking was studied using YASARA structure software version 23.12.24.W.64 over a period of 10.00 ns. After initial structural preparation (structural cleanup and pH adjustment), simulations were performed within YASARA, using the AMBER14 force field integrated into the YASARA structure (macro: md_run.mcr). To elaborate, the Posisson-Boltzmann approach is the technique chosen to manage the electrostatic force to obtain an electrostatic potential with an implicit solvent and counter ions. A “simulated cubic cell” was installed and flooded with water, maintaining a density of 0.997 g/mL and an isobaric pressure at 298 K. To mimic a neutral physiological environment, sodium chloride was introduced at a concentration of 0.9%. The distance between the protein and the cell boundary was set to 10 Å.

## Results and discussion

### *In-silico* study of Gallic acid and its derivatives (N-Alkyl Gallamide)

Drug-protein interactions were analyzed with the lead compound, which is gallic acid. Furthermore, analysis of the interaction of gallic acid with protein was done with the STITCH network server with a high confidence level of 0.700. A total of 157 nodes (the number of interacting proteins), 1923 edges (the number of associations formed), and four interactions of gallic acid with proteins were obtained. Table 1 shows the results of the analysis of the interaction of gallic acid with proteins at a high confidence level of 0.700. Visualization of gallic acid interaction with proteins using Cytoscape software with a circular display type. ^24^

**Table 1.** The results of the analysis of the interaction of gallic acid with protein.

No.	Gallic acid interaction with:	*Combined score*
1	JUN	0.823
2	CASP3	0.745
3	AKT1	0.742
4	CASP7	0.700

Gallic acid is the leading compound in this study and was used as a drug compound to be analyzed for protein-drug interactions. In addition, many studies related to gallic acid as an anticancer agent have been carried out; therefore, research data on gallic acid are widely available in the database. Due to this, it is presumed that the gallic acid derivatives (N-alkyl gallamides) have at least the same type of interaction as gallic acid. The interaction of gallic acid with breast cancer proteins and apoptosis resulted in four interactions ( Table 1). ^24^ The interaction of gallic acid with four proteins, namely JUN, CASP3, AKT1, and CASP7 had a high combined score. The combined score for each protein are JUN (0,823), CASP3 (0,745), AKT1 (0,742) and CASP7 (0,700). The combined score was obtained bas on the volume of research data in the database, which includes co-expression, co-occurrence, experiment, and text mining scores. The higher the combined score of the interaction, the higher the confidence level. This type of interaction between gallic acid and the target protein is an agonist interaction. Selected target proteins related to breast cancer and proteins that play a role in the process of apoptosis. The interaction of gallic acid with selected proteins is expected to activate the apoptosis process of breast cancer cells.

JUN (c-JUN) is a transcription factor 1 (AP-1) driving protein that binds and activates transcription on the TRE/AP-1 element. Growth factors such as extra or intracellular signals, changes in onco-proteins, and UV light exposure stimulate c-JUN phosphorylation at serine 63/73 and activate c-JUN-dependent transcription. Therefore, activated c-JUN has the potential to play an essential role in carcinogenesis and cancer development. ^25^

AKT, also known as protein kinase B, is a critical element of the PI3K/AKT signalling pathway. ^26^ In addition, AKT regulates cancer characteristics such as tumor growth, as well as survival and invasion of tumor cells. AKT has three different isoforms, those being AKT1, AKT2, and AKT3. The three isoforms are reported to have specific effects on breast cancer. AKT1 plays a role in early tumors, AKT 2 is responsible for tumor development and metastasis, while AKT3 is associated with negative ER (Estrogen Receptor) status. Moreover, AKT1 increases cell proliferation through cell cycle proteins such as 21, p27, and cyclin D, and plays a role in preventing apoptosis through p53. ^27^ AKT1 was also reported to be involved in regulating breast cancer progression tested *in vivo* in ErbBB-induced mice. In this study, AKT1-deficient breast epithelial tumor cells (MEC) were reduced in size and proliferative capacity with a reduced abundance of cyclin D1 and p27. ^28^

CASP3 and CASP7 are a group of proteins involved in the process of cell apoptosis. More precisely CASP3 and CASP7 are effector caspases along with CASP6. All caspase families are inactive zymogens (pro-caspases) and their activation requires proteolytic activation during apoptosis. Effector caspases are activated by initiator caspases (CASP2, CASP8, CASP9, and CASP10) through cleavage at internal Asp residues leading to disassembly of large and small subunits, whereas inhibitory caspases are activated by dimerization via signals obtained from death receptors. ^29^ In breast cancer, CASP3 is overexpressed and is significantly associated with poor breast cancer-specific survival and provides additional prognostic value in different phenotypes. ^30^ The expression of CASP7, which is a pro-apoptosis protein sterically inhibited by XIAP protein, causes inhibited apoptosis. Higher levels of CASP7 were found in well-differentiated tumors, including ER+ breast tumors. This is due to the presence of an estrogen receptor element located in the CASP7 promoter area. CASP7 expression was significantly associated with estrogen receptor (ERα) expression status and continued to increase at the breast tumor stage. ^31^

The KEGG pathway analysis based on the interaction of gallic acid with JUN, AKT1, CASP3, and CASP7 proteins indicated several signalling pathways, including the PI3K-AKT signalling pathway, MAPK signalling pathway, estrogen signalling pathway, receptor-mediated extrinsic pathway, and TNF-signalling pathway. These signalling pathways are related to cell proliferation, the cell cycle, and apoptosis. ^26^

Selected target proteins, JUN, AKT1, CASP3, and CASP7 are predicted to play a role in the breast cancer mechanism pathway as a therapeutic pathway for gallic acid and its derivatives summarized by the KEGG database. ^26^ There are four predicted pathways, namely estrogen signalling pathways, PI3K/AKT signalling pathways, extrinsic receptor mediated pathways, and TNF-α signalling pathways. In the estrogen-mediated pathway, gallic acid and its derivatives are expected to bind to JUN proteins that can inhibit JUN activity in the cell cycle process. Whereas in PI3K/AKT signalling pathways, when AKT has been activated then gallic acid and its derivatives in the cell membrane are expected to inhibit both AKT activity and the activity of a downstream protein, namely mTOR so that the process of cancer cell proliferation can be inhibited. In contrast to JUN and AKT, whose activity is expected to be inhibited by gallic acid and its derivatives, the extrinsic and TNF-α signalling pathways are expected to activate CASP3 and CASP7 to immediately carry out the apoptosis process. According to this description, the relationship between proteins that interact with gallic acid based on protein-drug interaction analysis has a role and function in breast cancer related to the growth, development, and apoptosis of breast cancer cells.

Breast cancer protein-associated pathways and apoptosis were obtained by enrichment analysis of the KEGG pathway using the Enrichr database ( https://maayanlab.cloud/Enrichr/). One hundred and sixty-nine KEGG pathways were enriched, including breast cancer pathways and deep cancer pathways. Each enriched p value was calculated and compared with the Fisher method, where a p value<0.01 was significantly enriched. The p values were ordered from smallest to largest. The top 10 ontology gene enrichment results are presented in underlaying data. ^32^

Genes that interact with gallic acid directly or indirectly were analyzed for enrichment of the KEEG pathway in order to explore biological pathways involving related genes. The top ten KEEG pathways were significantly enriched with p values<0.001, the pathway in breast cancer was the main biological pathway enriched in related genes. Other biologic pathways in the 10 enriched pathways are associated with cancer, from colorectal cancer to endometrial cancer. The genes involved in the biological pathway of breast cancer were then analyzed for gene ontology (GO) enrichment. GO is a technique for interpreting high throughput molecular data and generating hypotheses about biological phenomena that underlies experiments or interpreting data (genes) using the ontology classification system of genealogy. ^33^

The molecular interaction and reaction networks of cells are described in the KEGG pathway, a database of biological pathways. Pathway enrichment analysis and the identification of differentially expressed genes (DEGs) are two common bioinformatics research techniques. ^34^^–^^36^ KEGG pathway analysis can be used to find important genes that relate to prognosis in the context of identifying biomarkers. ^34^^,^^36^^,^^37^ For instance, KEGG pathway enrichment analysis on DEGs was carried out in a study on colorectal cancer to find pathways relating to extracellular matrix, epithelial cell proliferation, and cell adhesion. ^34^ Another thyroid cancer study employed KEGG pathway analysis to investigate the biological development and molecular roles of TME-related DEGs. ^35^ KEGG pathway and protein-protein interaction network analysis was coupled in a study on triple-negative breast cancer to uncover 7 genes that related to survival rate. ^37^ Finally, KEGG pathway analysis was employed in a study on depression to forecast gene phenotypes based on GO and KEGG pathway enrichment scores.

The functional enrichment analysis of GO proteins that play a role in breast cancer and the mechanism of apoptosis consists of three aspects: cell composition, biological processes, and molecular functions. The analysis was carried out through the Enrichr database ( https://maayanlab.cloud/Enrichr/). A total of 73 enrichment results we retrieved in aspects of cell composition, including the nucleus, intracellular membrane-bounded organelle, cyclin-dependent protein kinase holoenzyme complex, and other cell components. The enrichment results on aspects of biological processes showed that 1086 included negative regulation of the apoptosis process, positive gene expression regulation, fibroblast cell proliferation, and other biological processes. Meanwhile, the aspect of the molecular function illustrated 111 functions involving serine/threonine/tyrosine kinase protein activity, phosphatase binding, kinase binding, and other molecular functions. Each enriched p value was calculated and compared with the Fisher method, where p values<0.01 were significantly enriched. The p values were ordered from smallest to largest. ^38^

There are three categories in GO, namely biological processes, molecular functions, and cellular components. Biological processes refer to the biological goals to which genes or gene products contribute. The process is achieved by means of one or more ordered sequences of molecular functions. Processes often involve chemical or physical transformations. Molecular function is defined as biochemical activity including binding to specific ligands or structures of gene products. Molecular functions simply describe what is done without specifying where or when it occurs. Meanwhile, the cellular component refers to the place in the cell where the gene product is active. ^39^

In this study, the ontology gene enrichment of the genes inside the breast cancer pathway was significantly enriched with the biological process category called “negative regulation of the apoptosis process”. Negative regulation of the apoptosis process is defined as a series of gene products involved in the breast cancer pathway that inhibit apoptosis or ‘downregulate’ apoptosis. The pathway involved in this biological process is the Wnt canonical signalling pathway. Canonical Wnt signalling is the pathway that is responsible for β-catenin and T cell factor (TCF) or lymphoid enhancer factor (LEF), which is responsible for the proliferation and metastasis of breast cancer cells ^40^ and maintenance of stemness. ^41^ Wnt signaling in breast cancer is activated by loss of the TP53 gene. ^42^

Next, GO in the category of molecular function that was significantly enriched, specifically the biochemical activity of the enzyme “serine/threonine kinase”, will be discussed. Serine/threonine kinase is a member of the protein kinase superfamily that phosphorylates the amino acids serine or threonine. Through phosphorylation of protein kinases, it chemically transfers phosphate from ATP (Adenosine Triphosphate) or GTP (Guanosine Triphosphate) to targeted amino acids with the release of hydroxyl groups from their protein substrates. The phosphorylation process can induce conformational changes in the substrate protein which can disrupt protein-protein interactions. This conformational change influences its protein activity, cellular localization, or association with other proteins. These protein/enzyme kinases have been shown to regulate important molecular pathways in cellular processes including proliferation, metabolism, migration, survival, and apoptosis. ^43^ Uncontrolled kinase activity due to mutation or loss of inhibitory mediators are commonly found in cancers, including breast cancer.

In breast cancer, serine/threonine kinase activity, also known as protein kinase B/Akt, interacts with breast tumor kinase (Brk) or protein-tyrosine kinase, which is involved in growth and cell survival and is overexpressed in most breast cancers but not in normal breast epithelial cells. ^44^ Recently, one of the serine/threonine kinases (D) family, namely protein kinase D3 (PRKD3) was reported to promote the proliferation, growth, migration, and invasion of cancer cells in several types of tumors including breast cancer and it is said to be an alternative therapeutic target that is promising for cancer treatment. ^45^

The next category of GO to be discussed is the significantly enriched cellular component called “nucleus”. As previously explained, cellular components describe the sites in the cell where activities occur whereas gene products are active. In this study, many genes in the breast cancer pathway were involved in the process of cell proliferation and interact with transcription factors, where the site of the process was in the cell nucleus. In addition, the significantly enriched cellular component is “intracellular membrane-enclosed organelles” based on the definition of the ontology gene browser. These organelles are organized structures of distinctive morphology and function, bound by single or multiple lipid bilayer membranes and are found within cells. This includes the nucleus, mitochondria, plastids, vacuoles, and vesicles, but excludes plasma membranes. From this definition, there are several places in the cell that are the site of cell proliferation and apoptosis and support the objectives of this study regarding apoptosis agents.

Molecular docking analysis was used to computationally predict the interaction between proteins and the test compound (ligand). The target protein was selected based on the analysis of drug interactions with proteins with a high confidence value. These proteins include JUN (PDB ID: 2P33), AKT1 (PDB ID:1H10), CASP3 (PDB ID:3KJF), and CASP7 (PDB ID: 5V6U) taken from the PDB data bank ( http://www.pdb.org/). The protein structure that was used is a 3D crystal structure downloaded in PDB format.

AutoDock 4.2.6 software was used to prepare each protein. The water molecules were removed from the protein, and hydrogen and charge were added. The original protein and ligand were separated. The prepared proteins and ligands were subsequently stored as PDBQT files. The original protein and ligands were used to validate the grid box, which is a location for the attachment of gallic acid molecules and their derivatives. Validation of grid box dimensions began with 40x40x40, 50x50x50, and 60x60x60, or was adjusted to the original ligand size of each protein. The best validation results are chosen when the RMSD reference value is less than 2Å and the binding free energy (ΔG) is low ( Table 2).

**Table 2.** Grid Box Validations.

	ΔG (kcal/mol)		
*Grid Center*	40x40x40	50x50x50	60x60x60
Protein JUN			
X = 23.679	-8.82	-9.2	-9.3
Y = 9.022			
Z = 30.409			
*RMSD references Å*	0.46	5.2	5.39
*Inhibition Constant (*nM)	340.35	181.59	151.96
Protein AKT1			
X = 15.149	-15.93	-14.72	-14.76
Y = 24.335			
Z = 16.343			
*RMSD references Å*	1.46	2.1	1.35
*Inhibition Constant* (pM)	2.12	16.31	15.26
Protein CASP3			
X = -44.236	-8.47	-8.1	-8.97
Y = 9.243			
Z = -21.625			
*RMSD references Å*	1.63	3.88	1.51
*Inhibition Constant* (nM)	613.63	1.15 (μM)	265.29
Protein CASP7			
X = -42.505	-7.41	-7.55	-7.15
Y = 12.409			
Z = 10.242			
*RMSD references Å*	2.03	1.27	1.82
*Inhibition Constant* (μM)	3.7	2.9	5.76

Gallic acid and its derivatives, along with tamoxifen used as a control, were then used as test ligands for molecular modeling these proteins with the best grid box. The results of molecular docking are predictive values of binding free energy, inhibition constants, and bond interactions between test ligands and protein amino acid residues. The results of the molecular docking of 13 test compounds to four proteins are shown in Table 3.

**Table 3.** The results of the molecular docking analysis of gallic acid and N-alkyl gallamide.

No	Compounds	JUN		AKT1	
		ΔG (kcal/mol)	Ki (μM)	ΔG (kcal/mol)	Ki (μM)
1.	Gallic acid	-4.82	292.07	-7.80	1.93
2.	Tamoxifen	-7.42	3.65	-4.38	611.97
3.	N-Methyl Gallamide	-5.53	87.83	-5.86	50.89
4.	N-Ethyl Gallamide	-5.86	50.53	-5.99	40.80
5.	N-Propyl Gallamide	-6.16	30.47	-6.06	36.07
6.	N-Butyl Gallamide	-6.31	23.77	-5.96	42.60
7.	N-Sec-Butyl Gallamide	-6.40	20.43	-6.20	28.52
8.	N-Ters-Butyl Gallamide	-6.57	15.41	-6.51	17.02
9.	N-Amyl Gallamide	-6.51	16.78	-5.88	48.82
10.	N-Isoamyl Gallamide	-6.74	11.52	-6.23	27.34
11.	N-Hexyl Gallamide	-6.73	11.68	-5.82	54.28
12.	N-Heptyl Gallamide	-6.73	11.69	-5.39	111.51
13.	N-Octyl Gallamide	-6.99	7.46	-5.27	138.07

No	Compounds	CASP3		CASP7	
		ΔG (kcal/mol)	Ki (μM)	ΔG (kcal/mol)	Ki (μM)
1.	Gallic acid	-5.17	161.71	-6.45	18.78
2.	Tamoxifen	-6.34	22.63	-8.13	1.09
3.	N-Methyl Gallamide	-5.78	57.63	-6.24	26.64
4.	N-Ethyl Gallamide	-5.93	44.74	-6.40	20.45
5.	N-Propyl Gallamide	-5.81	54.73	-6.58	15.09
6.	N-Butyl Gallamide	-5.92	45.61	-6.87	9.16
7.	N-Sec-Butyl Gallamide	-6.15	30.92	-6.89	8.83
8.	N-Ters-Butyl Gallamide	-6.57	15.39	-6.67	12.95
9.	N-Amyl Gallamide	-6.20	28.37	-7.07	6.62
10.	N-Isoamyl Gallamide	-6.19	28.86	-7.27	4.72
11.	N-Hexyl Gallamide	-6.30	24.05	-7.09	6.34
12.	N-Heptyl Gallamide	-5.79	57.00	-6.66	13.19
13.	N-Octyl Gallamide	-6.05	36.47	-7.08	6.48

Next, the best five ranked compounds were selected based on the results of molecular anchoring with the smallest G and Ki values for each protein. A total of nine gallic acid derivatives were obtained which were candidates for further analysis, namely ADMET analysis. ADMET analysis uses the SwissAdme webserver, pkCSM, Marvin Sketch, and the ProtoxII Prediction webserver. Table 4 shows the predicted results of ADMET analysis of gallic acid and the nine gallic acid derivatives N-alkyl gallamide.

**Table 4.** ADMET analysis prediction of gallic acid and their derivatives (N-Alkyl gallamide).

Compound	Drug Likeness Lipinski					Adsorption		Distribution
	BM	HBA	HBD	Log P	TPSA	Gi Absorption	P-gp Substrate	Fraksi un-bound
(1)	170.12	11	4	0.50	97.99	43.37	No	0.617
(2)	197.19	4	4	0.58	89.79	72.86	yes	0.642
(3)	211.21	4	4	0.90	89.79	71.29	yes	0.611
(4)	281.35	4	4	2.64	89.79	90.44	yes	0.442
(5)	253.29	4	4	1.90	89.79	91.15	yes	0.515
(6)	267.32	4	4	2.23	89.79	90.79	yes	0.479
(7)	239.27	4	4	1.57	89.79	91.75	yes	0.550
(8)	197.19	4	4	0.58	89.79	90.71	yes	0.548
(9)	211.21	4	4	0.87	89.79	94.18	yes	0.699
(10)	225.24	4	4	1.13	89.79	93.75	yes	0.558

Compound	Distribution	Metabolism				Excretion	Toxicity	
		Inhibitor						
	BBB	CYP 2C19	CYP 2C9	CYP 2D6	CYP 3A4	Total Clearances	LD50	AOT
(1)	-1.102	No	No	No	No	0.518	2000	IV
(2)	-0.982	No	No	No	No	0.624	2000	IV
(3)	-1.016	No	No	No	No	0.310	2000	IV
(4)	-1.156	No	No	yes	No	0.978	2000	IV
(5)	-1.097	No	No	No	No	0.356	2000	IV
(6)	-1.125	No	No	No	No	0.373	2000	IV
(7)	-1.071	No	No	No	No	0.339	2000	IV
(8)	-0.794	No	No	No	No	0.216	2000	IV
(9)	-1.101	No	No	No	No	0.261	2000	IV
(10)	-0.0996	No	No	No	No	0.499	2000	IV

**Description**: **Compound** (1): Gallic acid, (2): N-Ethyl gallamide, (3): N-Profile gallamide, (4): N-Octyl gallamide, (5): N-Hexyl gallamide, (6): N-Heptyl gallamide, (7): N-Amyl gallamide, (8): N-Isoamil gallamide, (9): N-Sec Butyl gallamide, (10): N-Ters-butyl gallamide. **P-gp Substrate**: yes, indicates the compound is predicted to be P-gp substrate, no indicates the compound is not predicted to be P-gp Substrate. **CYP inhibitors**: yes, it shows that the compound can inhibit CYP inhibitors, but it doesn't show that the compound does not inhibit CYP inhibitors. **BM**: Molecular Weight, **HBA**: Hydrogen Bond Acceptor, **HBD**: Hydrogen Bond Donor, **TPSA:** Total Polar Surface Area, **Log P**: Octanol/water solubility **, Gi Absorption**: Gastro Intestinal Absorption, **Un-bound fraction**: unbound fraction of drug molecules. **BBB**: Blood Brain Barrier, **LD50**: Lethal Dose 50%, **AOT**: Acute of Toxicity.

Based on the log P characteristic profile which interprets the solubility of the octanol/water phase and the permeability of gallic acid compounds and their derivatives, it can be classified in the biopharmaceutical classification system (BCS) by following the classification system: class I (high solubility and high permeability), class II (low solubility and high permeability), class III (high solubility and low permeability), and class IV (low solubility and low permeability). Predictions of the biopharmaceutical classification system for gallic acid compounds and their derivatives (N-alkyl gallamides) are summarized in Table 5.

**Table 5.** Prediction of the Biopharmaceutical Classification System (BCS) for gallic acid compounds and their derivatives (N-Alkyl Gallamide).

Compound	Solubility		Permeability		BCS Class
	Log p	Level	Gi Absorption	Level	
Gallic Acid	0.50	High	43.37	High	I
N-Ethyl Gallamida	0.58	High	72.86	High	I
N-Propyl Galamida	0.90	High	71.29	High	I
N-Octyl Gallamida	2.64	High	90.44	High	I
N-Hexyl Gallamida	1.90	High	91.15	High	I
N-Heptyl Gallamida	2.23	High	90.79	High	I
N-Amyl-Gallamida	1.57	High	91.75	High	I
N-Isoamyl Gallamida	0.58	High	90.71	High	I
N-Sec Butyl Gallamida	0.87	High	94.18	High	I
N-Tert Butyl Gallamida	1.13	High	93.75	High	I

**Description:** Log P: Octanol/water Solubility, Gi Absorption: Gastro Intestinal Absorption.

In addition, the relationship between pH and Log D, which shows the distribution coefficient of gallic acid compounds and their derivatives, is determined. The purpose of this predictive analysis is to measure the lipophilicity of ionizable compounds, where the partition is a function of pH. Table 6 shows the predicted relationship between pH values and Log D of gallic acid compounds and their derivatives (N-alkyl gallamides).

**Table 6.** Prediction of the relationship between pH and Log D of gallic acid compounds and their derivatives (N-Alkyl gallamide).

pH	Log D									
	(1)	(2)	(3)	(4)	(5)	(6)	(7)	(8)	(9)	(10)
1.5	0.71	0.43	0.90	2.88	2.09	2.49	1.69	1.63	1.31	0.92
5	-0.38	0.43	0.90	2.88	2.09	2.49	1.69	1.63	1.31	0.92
6.5	-1.82	0.42	0.89	2.87	2.08	2.47	1.68	1.62	1.30	0.91
7.4	-2.55	0.34	0.81	2.79	2.00	2.40	1.60	1.54	1.22	0.83

**Description**: Compound (1): Gallic acid, (2): N-Ethyl gallamide, (3): N-Profile gallamide, (4): N-Octyl gallamide, (5): N-Hexyl gallamide, (6): N-Heptyl gallamide, (7): N-Amyl gallamide, (8): N-Isoamyl gallamide, (9): N-Sec Butyl gallamide, (10): N-Ters-butyl gallamide.

The selection of the five best ranked gallic acid derivative compounds was made based on its binding values of ΔG and Ki (ordered from highest affinity). Ligand and amino acid interactions were visualized using LigPlot+ and LiganScout software. The interactions formed between the ligands and the amino acid residues of each protein are in the form of hydrophobic interactions and hydrogen bond interactions. Each interaction formed on the test ligand was compared with the original ligand. An example of visualization of the interaction of amino acid residues with ligands using LigPlot+ and LigandScout can be seen in underlaying data. ^46^ Hydrophobic interactions are shown in red semicircles, while hydrogen bond interactions are denoted by green dashed lines. The numbers on the hydrogen bond indicates the hydrogen bond distance between the amino acid residue and the ligand in angstrom units (Å).

The absorption of drug compounds and evaluation of drug likeness properties can be predicted by Lipinski's five of rules. These rules describe the molecular properties which are important for the pharmacokinetics of drug candidates. The Lipinski’s data of the drug candidate provides information about its similarity as a ligand of the drug. A good drug candidate will have Lipinski's rule of five data as follows, it has a molecular weight less than 500 Da, it does not have a hydrogen bond acceptor (HBA) of more than 10, it does not have a hydrogen donor bond (HBD) of more than 5, it has a log P value of no more than 5, and it must have a total polar surface area (TPSA) less than 140. ^47^ A complete prediction of the ADMET characteristic profile is shown in Table 4.

ADMET is used to describe the absorption, distribution, metabolism, excretion, and toxicity of drugs. *In-silico* ADMET profiling is a useful tool for predicting the pharmacological and toxicological properties of drug candidates, especially at the pre-clinical stage. To improve the prediction of ADMET, an *in-silico* model has been applied. The use of these models in particular has contributed to drug optimization and the avoidance of late-stage failures, which is also important because such failures lead to unproductive investments of time and money.

The absorption is predicted based on the nature of solubility in water (Log P), lipophilicity, and absorption in the human intestinal tract (Gi absorption). Based on Table 1, the predicted solubility of Log P for all gallic acid compounds and their derivatives (N-alkyl gallamide) is less than 3. This shows that these compounds have good solubility. Log P also describes the role of compound hydration, the higher the log P value, the worse the hydration. The limit of a good log P value ranges from 2 to 3. ^48^ Predicted absorption in the human intestine of gallic acid compounds and their derivatives is high because they can absorb more than 30% of the compound. Gi absorption is also associated with the permeability of drug compounds in the body.

Distribution was predicted using P-glycoprotein (P-gp) substrate, blood-brain barrier (BBB) permeability, and non-segmentation-related descriptors. The descriptors were predicted using pkCSM-pharmacokinetics. P-gp is an ATP-dependent drug extraction pump and is found in various human tissues. All newly synthesized molecules must be P-gp substrates. According to the predicted results in this study, only gallic acid compounds were not predicted as p-gp substrates. The BBB is a complex structure that separates the central nervous system (CNS) from peripheral tissues. To maintain homeostasis in the CNS, the BBB controls the transfer of matter, nutrients, and cells from the blood to the brain and from the brain to the blood. It also participates in the clearance of cellular metabolites and toxins from the brain to the blood. ^48^ The BBB permeability values of our compounds were estimated to range from −0.0996 to −1.156.

The drug molecules in the plasma are in balance between being bound to serum proteins and unbound. The proportion of drug molecules in plasma that are not bound to proteins is called the “un-bound fraction”. This profile influences renal glomerular filtration and hepatic metabolism, resulting in values for the volume of distribution, total clearance, and drug efficacy. ^49^ The higher the binding of the drug to proteins in the blood, the less efficient it is to spread across cell membranes. In this study, the predictive value of the unbound fraction was large (from a scale of 0 to 1), which means that the binding of the drug to the protein in the blood is not too strong so that it can spread throughout the cell membrane well.

To make a candidate drug compound, it is also necessary to analyze the pharmacokinetics of the compound against cytochrome P450 (CYP) protein inhibitors. These proteins are marker proteins to determine the effect of the response of an anticancer drug and are metabolic enzymes in the liver. The human CYP enzymes important in drug metabolism are CYP 1A2, the CYP 2C family, CYP 2D6, and CYP 3A4. Therefore, in this study, we investigated whether gallic acid derivative compounds did not have an inhibitory effect on these enzymes. Based on Table 1 only N-octyl gallamide compounds had an inhibitory effect on the CYP2D6 enzyme.

Excretion occurs primarily as a combination of hepatic and renal clearance, is associated with bioavailability, and is important for determining dose levels to achieve steady-state concentrations. The predicted excretion values, using the pkCSM-pharmacokinetic total clearance descriptor, ranged from 0.216 to 0.978 ml/min/kg.

In addition, the toxicity of the drug compound was also analyzed to determine whether the drug compound is toxic or not, which aims to predict the level of safety for its use. The predicted toxicity profile in this study is AOT (acute of toxicity) which is related to LD50. AOT has several classes, namely class 1 (fatal if swallowed, LD50=5 mg/kg), class II (fatal if swallowed, 5<LD50 50), class III (toxic if swallowed, 50<LD50 300), class IV (harmful if swallowed, 300<LD50 2000), class V (possibly harmful if swallowed, 2000<LD50 5000), and class V1 (non-toxic, LD50>5000). ^50^ Based on this category gallic acid and its derivatives fall into class IV.

It has been discussed previously that the solubility and permeability profile of gallic acid and its derivatives (N-alkyl gallamide) are important in predicting the pharmacokinetic properties of drug candidates. Based on this, this study tries to classify these profiles into the biopharmaceutical classification system (BCS). Based on this classification, gallic acid and its derivatives are classified in class 1 BCS, because all compounds have high solubility and permeability ( Table 5).

Another important profile is the relationship between pH and Log D of gallic acid compounds and their derivatives. Log D is the distribution coefficient described for ionizable compounds because log D is a measure of the pH-dependent difference in solubility of all species in the octanol/water system. The importance of predicting the value of log D is to predict the permeability of drug compounds *in vivo.* The relationship with pH indicates that changes in the pH environment that may occur in orally administered compounds occur in the gastrointestinal tract. ^51^ This is because there is no constant pH in the body so pH is highly considered when predicting *in vivo* behavior of drug candidates. The pH range that is the primary focus of attention is pH 7.4, which is the physiological pH of blood serum, while the pH of the stomach is pH 1–3 and the ileum is pH 7–8. In this study, predictions of pH and log D values were made for gallic acid and its derivatives ( Table 6). Based on the prediction results, both gallic acid compounds and their derivatives have varying Log D values. Drug candidate compounds that have a high Log D value are highly lipophilic (Log D at pH 7.4>3.5) tend to have poor water solubility which can interfere with intestinal absorption. Based on this, the log D value of gallic acid compounds and their derivatives can be concluded to have good distribution coefficients as drug candidate 3,4,5-trihydroxy benzoics.

The determination of the five best gallic acid derivatives based on ADMET analysis could not be determined because all test parameters were met. The selection of five gallic acid derivatives was determined by ordering the binding energy values of G and Ki as a result of molecular docking analysis ( Table 3). Hydrogen bonding and hydrophobic bonding interaction between gallic acid derivative and amino acid residues in JUN, AKT1, and CASP3, CASP7 proteins is presented in Table 7 and visualized in underlaying data bellow. The number of hydrogen and hydrophobic bonds is compared with the original ligand interactions. In JUN proteins, in general, there are two hydrogen bonds in the amino acids Met149 and Asn152, this is different from the N-octyl gallamide compound which only has hydrogen bonds in Met149. However, the hydrophobic interactions of the N-octyl gallamide compound with JUN were more than other compounds. In addition, the JUN showed interaction with essential amino acid residues of gallic acid derivative compounds, namely Met149, Leu148, Lys93, Met146, Leu206, Val196, Ile70, and Val78.

**Table 7.** Analysis of the interaction of ligands with amino acid residues JUN, AKT1, CASP3 and CASP7.

a. Ligand interaction with amino acid residue of JUN						
Amino acid	Native ligand	(2)	(7)	(4)	(5)	(9)
Met49	√ (H: 2,77; 2,86)	√ (H: 3,02)	√ (H: 2,90)	√ (H: 3,06)	√ (H: 2,86)	√ (H: 3,19)
Leu148	√	√	√	√	√	√
Ala91	√	√	√	√	√	√
Glu147	√	√	√	√	√	√
Ile124	√	√	√	√	√	√
Lys93	X	√	x	X	√	X
Met146	√	√	√	√	√	√
Leu206	√	√	√	√	√	√
Val196	√	√	√	√	√	√
Asn152	√ (H: 2,85)	√	√ (H: 3,18)	√ (H: 3,17)	√ (H: 3,17)	√ (H: 3,06; 3,09)
Ala151	√	√	√	√	√	√
Asp150	√	√	√	√	√	√
Ile70	√	√	x	X	x	X
Asp207	X	√	x	X	x	X
Glu111	X	√	x	x	x	X
Val78	√	x	√	x	x	√
Gln155	√	x	x	x	x	X
Gly71	√	x	x	x	x	X
Ser72	√	x	x	x	x	X

b. Ligand interaction with amino acid residue of AKT1						
Amino Acid	Native Ligand	(9)	(7)	(8)	(2)	(1)
Glu17	√ (H: 2,83;3,16)	√ (H: 2,92)	√ (H: 2,95)	√ (H: 2,78)	√ (H: 2,87)	√ (H: 2,89)
Gly16	√	√	√	√	√	√
Lys14	√ (H: 2,64)	√	√	√	√	√
Arg25	√ (H: 2,91;3,33)	√ (H: 2,83)	√ (H: 2,88)	√ (H: 2,89)	√ (H: 2,84)	√ (H: 2,88)
Asn53	√ (H: 2,63;3,02)	√ (H: 3,06)	√ (H: 3,04)	√ (H: 3,08)	√ (H: 3,05)	√ (H: 3,06)
Arg23	√ (H: 2,76;3,08)	√	√ (H: 2,96;3,18)	√ (H: 3,00;3,34)	√ (H: 3,08)	√ (H: 3,05)
Ile19	√ (H: 2,95)	√	√	√	√	√
Tyr18	√	√	√	√	√	√
Arg86	√ (H: 2,89;2,93)	x	x	x	x	x
Phe55	√	x	x	x	x	x

c. Ligand interaction with amino acid residue of CASP3						
Amino Acid	Native Ligand	(9)	(4)	(6)	(7)	(8)
Trp206	√	√	√	√	√	√
Phe250	√	√	√	√	√	√
Ser249	√	√	√	√	√	√
Glu248	√	√ (H: 3,22)	√	√	√	√
Trp214	√	√	√	√	√ (H: 2,75)	√
Phe247	x	√	√	√	√	√
Asn208	x	√ (H: 3,03)	√ (H: 2,99)	√ (H: 2,96)	√	√ (H: 2,81)
Arg207	√ (H: 2,64; 2,77;2,90; 3,01)	√ (H: 2,72)	√	√	√ (H: 2,79)	√
Ser251	x	x	√	√	√	X
Phe256	√	x	√	√	x	X
Glu246	x	x	x	x	√	X
Tyr204	√	x	x	x	x	X
Ser205	√ (H: 2,62)	x	x	x	x	X

d. Ligand interaction with amino acid residue of CASP7						
Amino Acid	Native Ligand	(7)	(4)	(3)	(6)	(8)
Arg167	√ (H:2,90)	√	√	√	√	√
Phe219	√	√	√	√	√	√
Glu216	x	√	√	√	√	√
Lys160	√ (H:2,79)	√	√	√	√	√
Ile159	√	√	√	√	√	√
Asn148	x	√	√	√	√	x
Tyr223	√	√	√	√	√	√
Phe221	√	√	√	√	√	√
Thr163	√	√ (H: 2,94)	√ (H: 3,07)	√ (H: 3,13)	√ (H: 2,99)	√ (H: 2,75)
Ala164	√	√	√	√	√	√
Pro158	x	x	x	√	x	x
Met294	√	x	x	√	x	x

**Description**: (1) N-Ethyl gallamide, (2) N-Propyl gallamide, (3) N-octyl gallamide, (4) N-Hexyl gallamide, (5) N-Hepthyl gallamide, (6) N-Amyl gallamide, (7) N-Isoamyl gallamide, (8) N-Sec-Butyl gallamide, (9) N-Ters-Butyl gallamide. (√) no interaction, (X) no interaction.

In the AKT1 protein, the hydrogen bonding interactions on the original ligand were more than in the test compound. There were seven hydrogen bonds in the original ligand and three hydrophobic interactions, while the N-ters-butyl gallamide compound has three hydrogen bonds (Glu17, Arg25, Asn53) and five hydrophobic interactions. While the other four compounds have four hydrogen bonds and hydrophobic bonds, with hydrogen bonds at the same amino acid residues (Glu17, Arg25, Asn53, Arg23). Of all the interacting amino acids, there were only two essential amino acids that interacted with the test compound on the AKT1 protein, namely Lys14 and Ile19.

In CASP3 protein, the N-ters-butyl gallamide compound had more hydrogen bonds with amino acids than the original ligand and four other compounds. Amino acids that have hydrogen bonding interactions in N-ters-butyl gallamide compounds are Glu248, Asn208, and Arg207. Meanwhile, the essential amino acids that interact with the test compound on CASP3 protein are Trp206, Trp214, Phe250, Phe247, and Phe256. The essential amino acid residue that is hydrogen bonded with the N-isoamyl gallamide compound is Trp214.

In CASP7 protein, hydrogen bond interactions in the native ligand were more than that of the test compound. But uniquely, hydrogen bonds are formed on the same amino acid residues in all test compounds, namely Thr163, which is one of the essential amino acids. The hydrophobic bond interactions formed were also generally the same for all test compounds. The interacting essential amino acids include Phe219, Lys160, Ile159, Phe221, Thr163, and Met294.

Based on the study of the interaction of the test compounds with each target protein, the types of interactions formed are mostly similar to the original ligands for each protein. It can be stated that the test compound is attached to each protein in the same coordinates as the original ligand. In addition, the test compound for gallic acid and its derivatives is expected to have the same activity as the original ligand on each target protein.

*In-silico*, the selection of the best compounds derived from gallic acid as candidates for apoptosis agents involves several stages of selection. First, the designed gallic acid derivatives were subjected to molecular docking analysis with the selection criteria being the order of low bond energy (ΔG). The lowest (most negative) binding energies were ranked and the best 5 compounds were selected from each target protein. Second, the five best gallic acid derivatives based on the results of molecular docking analysis were selected based on the characteristic profile of ADMET, with the selection criteria being compounds that have a good ADMET characteristic profile and are non-toxic. However, in this study, the selection based on the characteristic profile of ADMET could not be determined, because gallic acid derivative compounds have good ADMET profiles as drug candidates.

Based on the results of molecular docking and ADMET profiling, the best of three gallic acid derivatives, namely N-octyl gallamide, N-ters-Butyl gallamide and N-isoamyl gallamide have a potency to be further developed as a promising anti-breast cancer agent.

### *In-vitro* study of gallic acid and its derivatives (N-Alkyl gallamide): Cytotoxic activity

**Figure 1.**
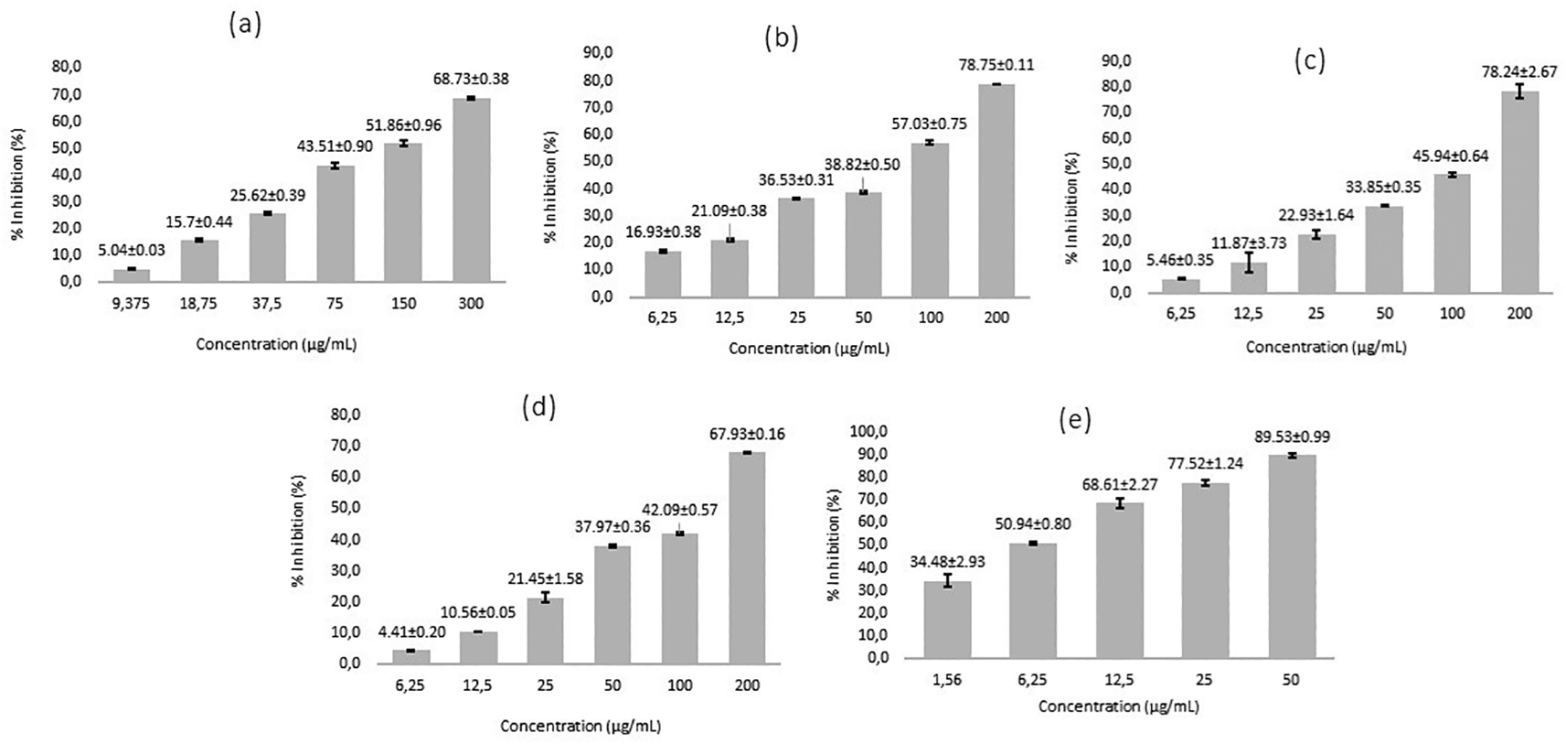
The relationship between percent inhibition of cell growth (% inhibition) against compound concentration. Inhibition percentage average ± Standard deviation (a) Gallic acid, (b) N-Octyl gallamide, (c) N-Tert-butyl gallamide, (d) N-Isoamyl gallamide, (e) Tamoxifen. % inhibition is proportional to the increase compound concentration.

Three gallic acid derivatives, namely N-Octyl gallamide, N-Tert-butyl gallamide, and N-Isoamyl gallamide, have been synthesized by Marcelia *et al.* (2018) ^19^ and Chan *et al.* (2018). ^20^ A cytotoxicity test was carried out using the MTT method [3-(4,5-dimethylthiazol-2-yl)-2,5-diphenyltetrazolium bromide]. Cytotoxic activity of gallic acid derivatives using the MCF7 cell line, which is a breast cancer cell line Gallic acid was used as a comparator compound, while tamoxifen was used as a positive control compound. The inhibitory concentration of 50%, or IC _50_, is the output of this analysis. The IC _50_ value was obtained by making a log relationship between the concentration of the sample and the percentage inhibition of each concentration. The percentage inhibition of cell growth is proportional to the concentration of compounds used. Percentage inhibition of each compound in proportion to the concentration of the compound used in the cells ( Figure 1).

The IC _50_ value of each compound is obtained from the linear line equation y=ax+b where y is the % inhibition and x is the log of concentration. To get the IC _50_ value, substitute the value y=50 to get the log x value, so that the value of x can be obtained by conversion to the anti-log x, which is the concentration of inhibition of cell growth as much as 50% in units μg/mL which is converted to μM after multiplied by the molecular weight each compound. The absorbance and linear equation curves are presented in underlying data. ^56^ IC _50_ values are summarized in Table 8.

**Table 8.** Cytotoxic activity of gallic acid derivatives in MCF7 cells.

No	Compounds	IC _50_ (μM)
1	Gallic Acid	703.4 ± 0.51
2	Tamoxifen	25.79 ± 0.08
3	N-Octyl gallamide	205.2 ± 0.44
4	N-Tert-Butyl gallamide	372.6 ± 4.09
5	N-Isoamyl gallamide	441.7 ± 1.41

Note: The IC _50_ value is the mean value of 3 repetitions of the experiment ± SD (standard deviation).

The MTT Assay is a method used to analyze the cytotoxic effect of a compound on cells. The principle of the MTT assay is the reduction reaction of MTT tetrazolium to formazan crystals, which is based on the ability of cellular oxidoreductase enzymes that depend on nicotinamide adenine dinucleotide (NADPH) to reduce tetrazolium MTT dye into insoluble formazan, which has a purple color. ^52^ Formazan will be formed from living cells with an active metabolism. When cells die, they will lose the ability to convert MTT into formazan. The more cells that live, the more formazan is formed. In this study, the higher the concentration of the test compound given to the cell, the less formazan is formed, the purple color fades, and the absorbance is small.

Gallic acid derivatives (N-Alkyl gallamide) have cytotoxic activity against the MCF7 breast cancer cell line. Research shows that the higher the concentration of the test compound, the higher the percentage of inhibition of cell growth (% inhibition) in a linear manner. Figure 1 shows the different levels of inhibition of each test compound. Tamoxifen, as a breast cancer control compound, has been shown to have a higher inhibition rate in MCF7 cells compared to gallic acid and gallic acid derivatives. In this study, an inhibitory concentration of 50% (IC _50_ value) was determined to express the cytotoxic activity of gallic acid derivatives against MCF7 breast cancer cells. The lower the IC _50_ value, the higher the cytotoxic activity. As shown in Table 8, compared to gallic acid, three gallic acid derivatives (N-Octyl galamide, N-Tert-Butyl gallamide, and N-Isoamyl gallamide) showed higher cytotoxic effects. However, compared to tamoxifen, these three compounds have a lower cytotoxic effect.

The high cytotoxic activity of the three compounds derived from gallic acid respectively are N-Octyl gallamide, N-Ter-butyl gallamide, and N-Isoamyl gallamide. N-Octyl gallamide has a higher cytotoxic activity compared to the other two gallic acid derivatives; this implies that the addition of a methylene group (-CH _2_) to the alkyl chain of the N-Alkyl gallamide compound can increase its hydrophobicity. This is supported by the research of Chan *et al.* (2018), ^20^ who reported that compounds derived from gallic acid that have long alkyl chains show anticancer activity against colorectal cancer. *In-vitro* test results also showed that the compound N-Tert-butyl gallamide had higher activity and cytotoxicity compared to N-Isoamyl gallamide. This implies that the many branches of the aliphatic carbon chain can increase its cytotoxic activity against MCF7 breast cancer cells. The results of this study are supported by research conducted by Notash B (2013), who reported that complex compounds with many branched chains will provide a steric effect that can increase their cytotoxicity against breast cancer. ^53^

Based on the 50% inhibitory concentration (IC _50_ value) obtained from the results of this study, the cytotoxic effect of the test compounds was significantly different when compared to the inhibition constant (Ki) on the predicted results of molecular docking. Gallic acid, tamoxifen, and the three gallic acid derivatives (N-Octyl gallamide, N-Tert-butyl gallamide, and N-Isoamyl gallamide) had a lower cytotoxic effect on MCF7 breast cancer cells than predicted by molecular binding. Nevertheless, in this study, the cytotoxic effect of gallic acid derivatives was better than that of the lead compound, gallic acid. The study conducted by Aborehab *et al.* (2021) ^54^ reported that gallic acid had a low cytotoxic effect on MCF7 cells with an IC50 of 7334.6 g/mL or equivalent to 43112 μM. This is significantly different from the results of this study.

### Gallic acid derivative compounds can induce apoptosis in MCF7 breast cancer cells

**Figure 2.**
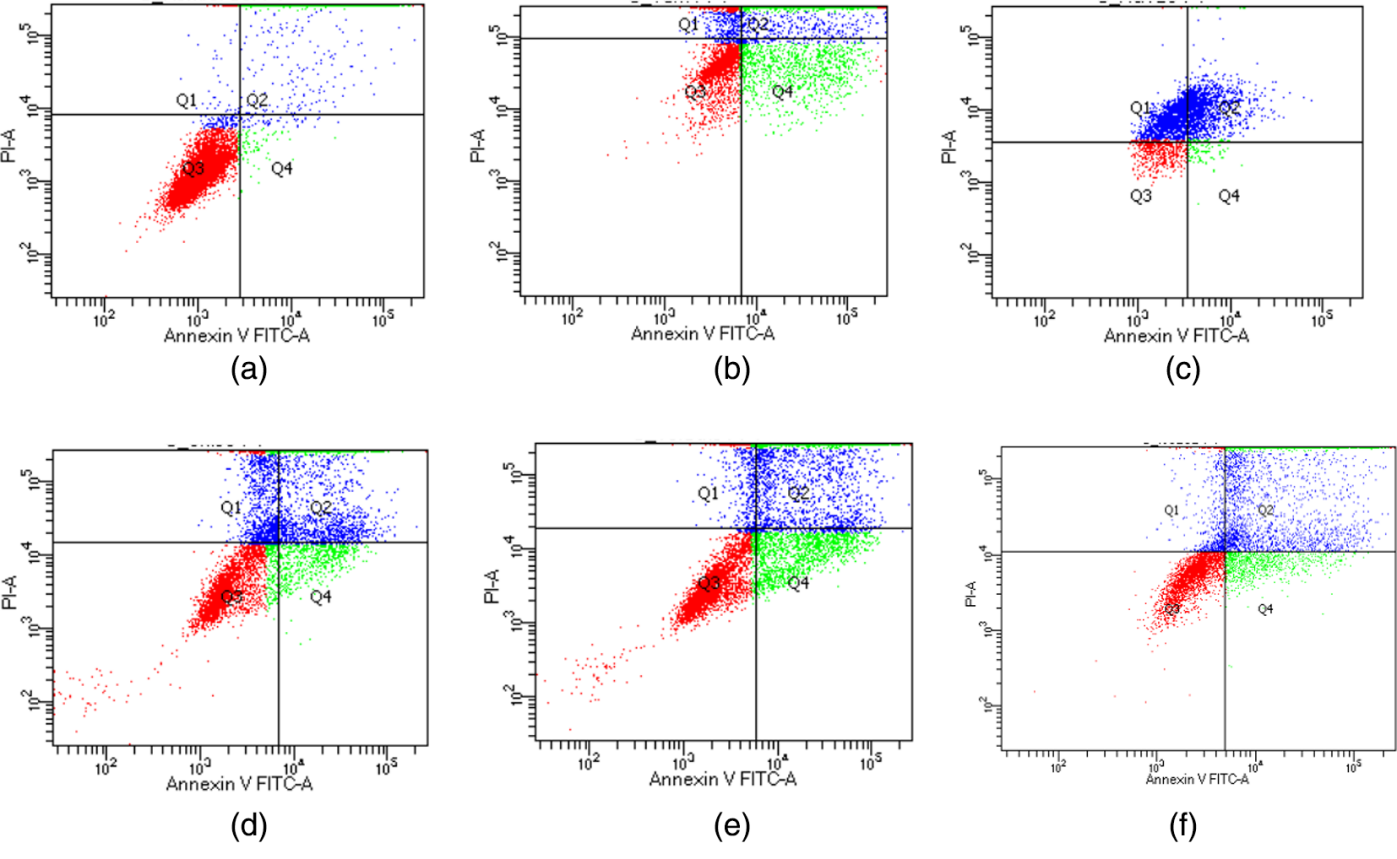
Plot of staining results of MCF7 cells with Annexin V/FITC/PI dyes Dot plots represent the four quadrant (Q) images captured by flow cytometry analysis. Q1, Necrotic Cells; Q2, Late Apoptosis Cells; Q3, normal cells; Q4, early apoptosis cells. (a) Control group of MCF7 cells; (b) Tamoxifen group; (c) Gallic acid group; (d) N-Octyl galamide group; (e) N-Tert-Butyl galamide group; (f) N-Isoamyl galamide group.

The IC _50_ value of gallic acid and its derivatives on MCF7 cells was known, then apoptosis activity was tested using double staining of Annexin V/FITC and PI to identify cells that underwent early apoptosis (early apoptosis/annexin V-positive and PI-negative) and cells that underwent late apoptosis (late apoptosis/annexin V-positive and PI-positive). MCF7 cells treated with gallic acid derivatives showed the highest proportion of cells at the late apoptosis stage compared to control MCF7 cells ( Table 9, Figure 2). Based on these results MCF7 cells treated with all samples including gallic acid and tamoxifen showed that gallic acid samples and their derivatives inhibited cell proliferation through induction of apoptosis reaching more than 50%.

**Table 9.** Apoptosis percentage of MCF 7 cells.

Compounds	Total (%)	Early apoptosis (%)	Late apoptosis (%)	Necrosis (%)
MCf7 cells Control	14.8	1.4	12.6	0.8
Tamoxifen	56.8	16.4	25.7	14.7
Gallic Acid	64.6	21.8	34.6	8.2
N-Octyl gallamide	55.9	10.5	25.3	20.1
N-Ters-Butyl gallamide	56.0	19.5	30.0	6.5
N-Isoamyl gallamide	55.8	12.4	35.0	8.4

The effect of gallic acid derivatives on apoptosis of MCF7 breast cancer cells ( Table 9; Figure 2) showed an increase in the ratio of cell death between early and late apoptosis. The population of cells that undergo necrotic death is lower than that of early and late apoptosis cells. Based on the analysis of the apoptosis of MCF7 cells, gallic acid and its derivatives have been shown to induce apoptosis. This can be seen from the significantly different total cell death values when compared to control MCF7 cells. Gallic acid can induce apoptosis with a higher percentage of cell death (64.6%) compared to its derivatives (N-Octyl gallamide, N-Tert-butyl gallamide, and N-Isoamyl gallamide). N-Alkyl gallamide compounds (N-Octyl gallamide, N-Tert-butyl gallamide, and N-Isoamyl gallamide) were proven to be able to induce the total population's death by apoptosis at a concentration of IC _50_ (μM) of 55.8 to 56.0%. Interestingly, these proportions showed that the apoptosis effects of the three compounds were not significantly different. In terms of cytotoxicity analysis, the N-Octyl gallamide compound had a better inhibitory proportion than the other two compounds.

In addition, the compound N-Octyl gallamide with the highest cytotoxic effect among the three had an apoptosis effect with the highest proportion of cell death due to necrosis, which was 20.1%. This can be caused by the influence of other factors outside the cell, such as hypoxia, extreme temperatures, the presence of toxins, physical trauma, and the presence of a viral infection, which is a feature of cell death by necrosis. ^55^ In this regard, it is necessary to conduct further research to determine the causal factors. In this study, the apoptosis effect of gallic acid is supported by a recent study by Aborehab *et al.* (2021), ^54^ who reported that gallic acid has an apoptosis effect of 11.58%, which is a smaller percentage of deaths due to apoptosis than this study.

### Molecular dynamic simulation

To investigate the dynamic stability of the CASP3-N-Alkyl gallamide complex (specifically N-Octyl gallamide, N-Isoamyl gallamide, and N-tert-butyl gallamide), molecular dynamics simulations were conducted. Utilizing YASARA, complex interactions were confirmed throughout a 10 ns simulation within an explicit solvation system. The assessment of complex interactions involved the calculation of various parameters such as RMSD, RMSF, hydrogen bond, and gyration radius values.

**Figure 3.**
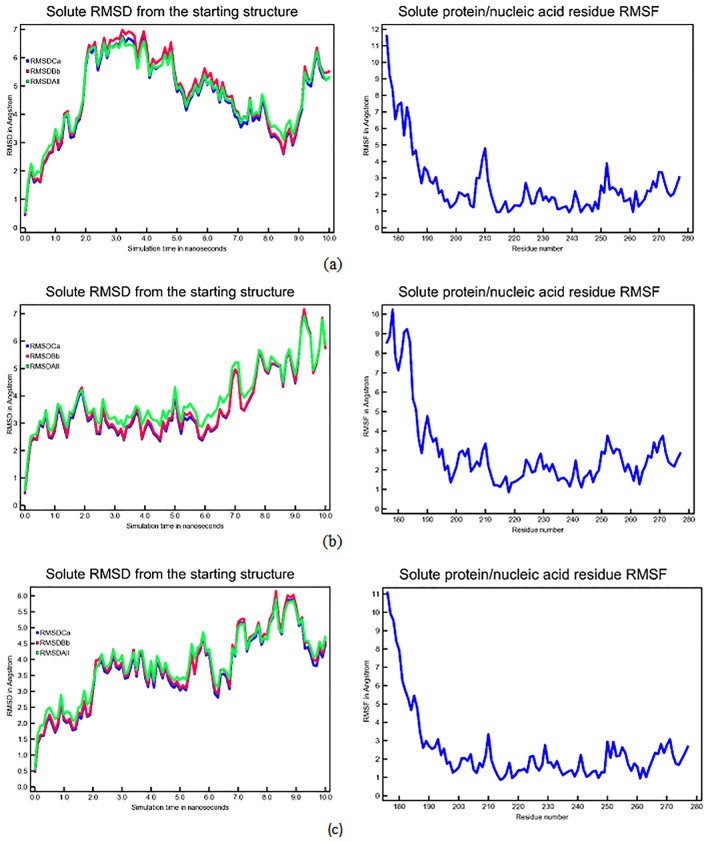
Dynamics analysis of CASP3-N-Alkyl Gallamide complex with 10 ns simulation. Left: Graph displays the mean square deviation (RMSD) of Cα atoms (blue), backbone atoms (red), and the mean RMSD (green); Right: Root mean square fluctuations (RMSF). (a) CASP3-N-Octyl Gallamide Complex; (b) CASP3-N-Ters Butyl Gallamide Complex; (c) CASP3-N-Isoamyl Gallamide Complex.

The RMSD curve serves as a deviation metric, illustrating the comparison between the ligand-receptor conformation during the ongoing simulation and the initial ligand-receptor conformation. A stable RMSD curve exhibits a consistent movement pattern over a specific period. Figure 3 depicts the RMSD and RMSF curves of the CASP3-N-Alkyl gallamide complex.

Analyzing the RMSD curves of the gallic acid derivative test compound for each receptor revealed stability throughout the 10 ns simulation. The RMSF curve, indicating the fluctuation of amino acid residues comprising the receptor during the simulation, reflects the flexibility of these residues. A high RMSF value signifies increased flexibility, potentially hindering amino acid-ligand binding. In this study, the RMSF values were satisfactory, showing fluctuations in essential amino acids of less than 2 Angstroms.

**Figure 4.**
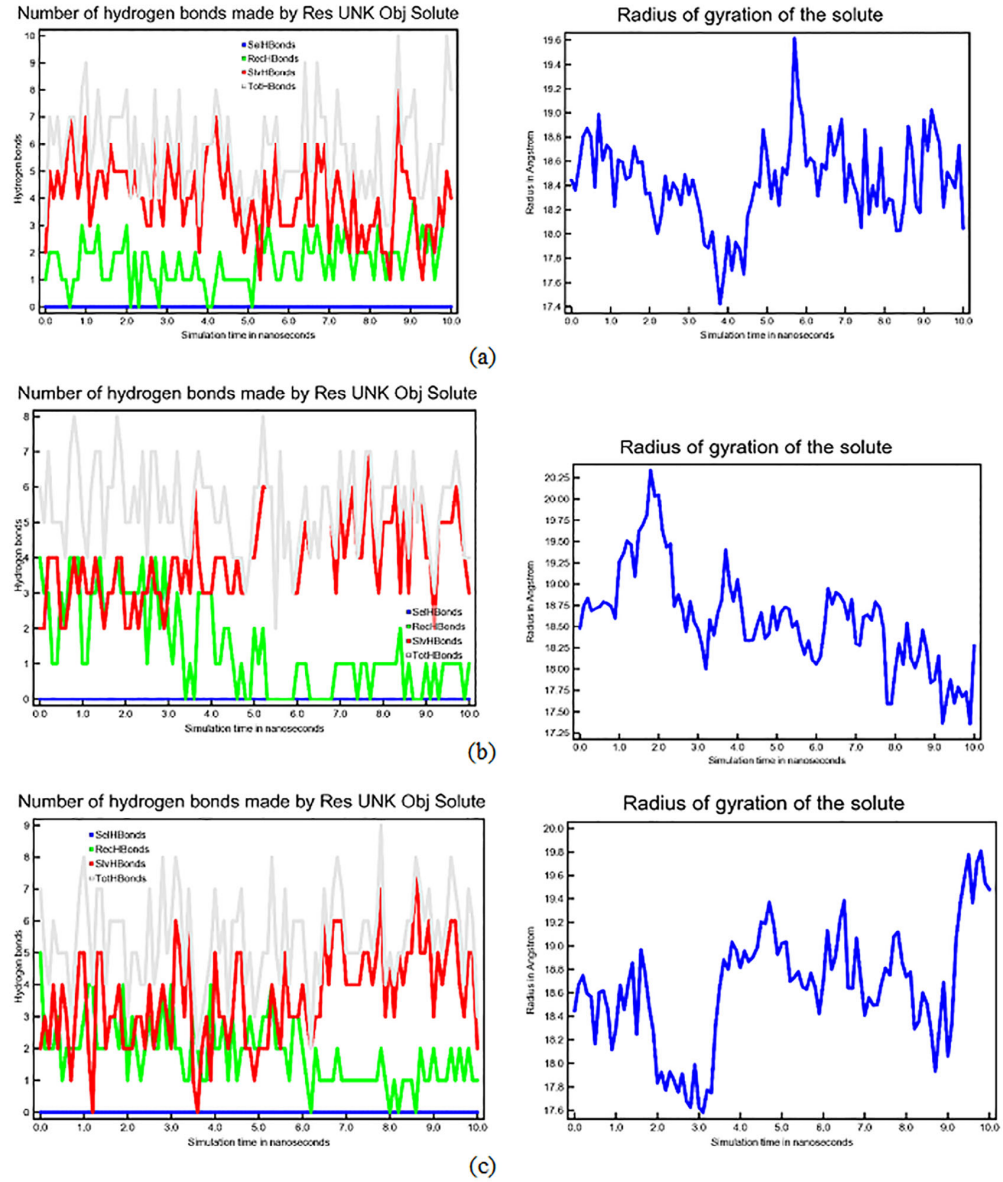
Interaction analysis through the number of hydrogen bonds and radius of gyration. Left: Number of hydrogen bonds is categorized as follows: intrinsic molecular hydrogen bonds are denoted in blue, bonds formed with the adjacent Complex CASP3-N-Alkyl Gallamide are green and those engaged with the aqueous environment are represented in red. Cumulative hydrogen bonds are depicted in gray. Right: The radius of gyration of CASP3-N-Alkyl Gallamide complex corresponds to 10 ns molecular dynamics simulation at 298 K; (a) CASP3-N-Octyl Gallamide Complex; (b) CASP3-N-Ters Butyl Gallamide Complex; (c) CASP3-N-Isoamyl Gallamide Complex.

In addition, the count of hydrogen bonds in the molecular dynamics simulation of the CASP3-N-Alkyl gallamide complex remains consistent throughout the 10 ns duration. Figure 4 illustrates these interactions as remarkably steady, while the radius of gyration indicates that the complex exhibits consistent stability with minimal structural movement over the 10 ns timeframe. Based on the results of the molecular dynamics simulation, the three N-Alkyl gallamide compounds demonstrated notable stability within the 10 ns simulation period. Nevertheless, a more in-depth analysis through molecular dynamics simulations with an extended timeframe, such as 100 ns, is still necessary to ensure and validate the stability.

At the end of this discussion, we can provide an opinion that the implications of this study are understanding disease mechanisms, targeted drug development, personalized medicine, drug resistance and biomarker discovery. In protein-protein interactions and drug protein interactions study play a crucial role in the development and progression of cancer. By studying these interactions, we can gain insight into the underlying molecular mechanisms of cancer and identify potential targets for therapeutic interventions. We can serve as potential targets for drug development. By identifying specific interactions involved in cancer progression, researchers can design drugs that disrupt these interactions and inhibit tumor growth. Studying protein-protein interactions and drug-protein interactions can help in the development of personalized treatment strategies for cancer patients. By analyzing the specific interactions present in an individual's tumor, researchers can tailor treatment approaches to target the unique molecular characteristics of the tumor. Besides that, this study can help researchers identify mechanisms of resistance and develop strategies to overcome it, improving the effectiveness of cancer therapies. Also, can serve as potential biomarkers for cancer diagnosis, prognosis, and treatment response. By studying these interactions, researchers can identify specific protein complexes or networks that are associated with different cancer types or stages, providing valuable information for clinical decision-making. There is a correlation between the predictive test using *in-silico* molecular modeling and the *in-vitro* MTT Assay confirmation test and apoptosis test, indicating that the predicted activity of the compounds N-Octyl gallamide, N-tert-butyl gallamide, and N-Isoamyl gallamide suggests their potential as agents for inducing breast cancer apoptosis.

## Conclusion

Based on *in silico* studies, it can be concluded that gallic acid and its derivatives (N-alkyl gallamide) can interact agonically with several proteins related to breast cancer and the process of apoptosis. The proteins with the highest confidence scores were JUN, AKT1, CASP3, and CASP7. Molecular docking between gallic acid and its derivatives (N-octyl gallamide, N-tert-butyl gallamide, and N-Isoamyl gallamide) indicates that there is a strong interaction and bond with these proteins so that they can potentially have good activity. The ADMET profile shows that gallic acid and its derivatives (N-octyl gallamide, N-tert-butyl gallamide, and N-Isoamyl gallamide) can be categorized as safe to be used as apoptosis agents in the future. Based on in vitro studies, it can be concluded that gallic acid derivatives (N-octyl gallamide, N-tert-butyl gallamide, and N-Isoamyl gallamide) can inhibit the growth of MCF7 breast cancer cells by 50% by inducing apoptosis.

## Data Availability

Figshare: 2D chemicals structure of gallic acid and it derivatives (N-Alkyl gallamide). https://doi.org/10.6084/m9.figshare.21391545.v1. ^22^ Figshare: Interaction of gallic acid with protein. https://doi.org/10.6084/m9.figshare.21391902.v1. ^24^ Figshare: Systematic prediction of four target proteins and gallic acid therapeutic pathways. https://doi.org/10.6084/m9.figshare.21391524.v1. ^26^ Figshare: Top 10 KEGG pathway enrichment analysis results. https://doi.org/10.6084/m9.figshare.21392082.v1. ^32^ Figshare: Top 10 ontology gene enrichment analysis results. https://doi.org/10.6084/m9.figshare.21392232.v1. ^38^ Figshare: Visualization of Ligand Interactions with Protein Amino Acid Residues. https://doi.org/10.6084/m9.figshare.21392520.v1. ^46^ Biostudies: Raw data gene list from KEGG with accession number S- BSST934. ^21^ Figshare: The Absorbance and Linear Equation Curves of MTT Assay Gallic Acid and Its Derivatives. https://doi.org/10.6084/m9.figshare.23702403. ^56^ Data are available under the terms of the Creative Commons Zero “No rights reserved” data waiver (CC0 1.0 Public domain dedication).
