# Supplementary material for: Molecular docking, ADMET profiling of gallic acid and its derivatives (N-alkyl gallamide) as apoptosis agent of breast cancer MCF-7 Cells
Source: F1000Res. 2024 Feb 8;11:1453. Originally published 2022 Dec 8. [Version 3] doi: 10.12688/f1000research.127347.3 (PMC10924730; doi:10.12688/f1000research.127347.3)
Supplement: Peer review history

# Reviewer report: Reviewer response for version 3

**DOI:** 10.5256/f1000research.162222.r245187

**Published:** 2024-03-09

**Authors:** Naveen Dhingra

**Affiliations:** 1 Science, Sanjeev Agrawal Global Educational University, Bhopal, Madhya Pradesh, India

The manuscript could be accepted

Is the work clearly and accurately presented and does it cite the current literature?

Yes

If applicable, is the statistical analysis and its interpretation appropriate?

Partly

Are all the source data underlying the results available to ensure full reproducibility?

Yes

Is the study design appropriate and is the work technically sound?

Partly

Are the conclusions drawn adequately supported by the results?

Yes

Are sufficient details of methods and analysis provided to allow replication by others?

Yes

Reviewer Expertise:

Systems Biology; Drug Designing; Cancer Biology; QSAR; Molecular Docking

I confirm that I have read this submission and believe that I have an appropriate level of expertise to confirm that it is of an acceptable scientific standard.

# Reviewer report: Reviewer response for version 2

**DOI:** 10.5256/f1000research.153995.r220298

**Published:** 2023-11-10

**Authors:** Naveen Dhingra

**Affiliations:** 1 Science, Sanjeev Agrawal Global Educational University, Bhopal, Madhya Pradesh, India

In the methodology the authors have used the protein after removing the water molecules whereas, in the mentioned protein the water molecules play very important role. So authors should perform the docking again after incorporating the water molecules.

 Authors have also not validated the software.

 In the figure 1 the standard deviation or error is not mentioned.

 In result and discussion section authors have not mentioned the result and its significance appropriately.

Is the work clearly and accurately presented and does it cite the current literature?

Yes

If applicable, is the statistical analysis and its interpretation appropriate?

Partly

Are all the source data underlying the results available to ensure full reproducibility?

Yes

Is the study design appropriate and is the work technically sound?

Partly

Are the conclusions drawn adequately supported by the results?

Yes

Are sufficient details of methods and analysis provided to allow replication by others?

Yes

Reviewer Expertise:

Systems Biology; Drug Designing; Cancer Biology; QSAR; Molecular Docking

I confirm that I have read this submission and believe that I have an appropriate level of expertise to confirm that it is of an acceptable scientific standard, however I have significant reservations, as outlined above.

# Reviewer report: Reviewer response for version 2

**DOI:** 10.5256/f1000research.153995.r199317

**Published:** 2023-08-31

**Authors:** Damilohun Samuel Metibemu

**Affiliations:** 1 Department of Chemistry, Physics, and atmospheric science, Jackson State University, Jackson, Mississippi, USA; 2 Department of Biochemistry, Adekunle Ajasin University,, Akungba-Akoko, Nigeria

The authors have addressed all my concerns

Is the work clearly and accurately presented and does it cite the current literature?

Yes

If applicable, is the statistical analysis and its interpretation appropriate?

Partly

Are all the source data underlying the results available to ensure full reproducibility?

Yes

Is the study design appropriate and is the work technically sound?

Partly

Are the conclusions drawn adequately supported by the results?

Partly

Are sufficient details of methods and analysis provided to allow replication by others?

Yes

Reviewer Expertise:

NA

I confirm that I have read this submission and believe that I have an appropriate level of expertise to confirm that it is of an acceptable scientific standard.

# Reviewer report: Reviewer response for version 1

**DOI:** 10.5256/f1000research.139846.r175977

**Published:** 2023-07-04

**Authors:** Damilohun Samuel Metibemu

**Affiliations:** 1 Department of Chemistry, Physics, and atmospheric science, Jackson State University, Jackson, Mississippi, USA; 2 Department of Biochemistry, Adekunle Ajasin University,, Akungba-Akoko, Nigeria

1. it is recommended that the authors define the full meaning of PDB, SDF, and PDBQT at the point of first use and for all the abbreviations.
2. What is the full meaning of ADMET? Please provide the meaning at the time of first use
3. The authors should explain what type of interactions gallic acid and its derivatives make with the protein. Are they inhibitors or agonists?
4. In addition to discussing the relevance of the combined scores on the KEGG pathway, the authors should provide references to other studies related to this study. A description of the KEGG pathway is not sufficient.
5. The authors are kindly requested to discuss the implications of this study for the different proteins and genes. What are the implications of this study for oncology?
6. Molecular docking and ADMET profiling results should be discussed in detail, including the implications of each value for potential anticancer compounds of gallic and its derivatives.
7. It is noteworthy that in-silico predictions are often not accurate, especially in clinical trials. In order to confirm the results obtained from in-silico screening, I recommend that the authors conduct some in-vitro admit profiling.
8. There is too much vagueness in the conclusion. A conclusion should be drawn based on the results of the study.

Is the work clearly and accurately presented and does it cite the current literature?

Yes

If applicable, is the statistical analysis and its interpretation appropriate?

Partly

Are all the source data underlying the results available to ensure full reproducibility?

Yes

Is the study design appropriate and is the work technically sound?

Partly

Are the conclusions drawn adequately supported by the results?

Partly

Are sufficient details of methods and analysis provided to allow replication by others?

Yes

Reviewer Expertise:

Bioinformatics, Cancer Biology, Medicinal Chemistry, and phyto-medicine

I confirm that I have read this submission and believe that I have an appropriate level of expertise to confirm that it is of an acceptable scientific standard, however I have significant reservations, as outlined above.

# Response

**Published:** 2023-07-13

**Authors:** Norma Nur Azizah

**Affiliations:** Universitas Indonesia, Indonesia

Thank you for reviewing our article. I have read all the reviews you have provided. We are trying to improve our article according to the review you provided.

 Best Regard,

 Norma Nur Azizah

# Response

**Published:** 2023-07-31

**Authors:** Norma Nur Azizah

**Affiliations:** Universitas Indonesia, Indonesia

To reviewers:

 Thank you for reviewing our article, we have tried to improve the article according to the suggestions for improvement that have been submitted. The following is a report on the response from the review team that we can provide, hopefully it will be well received.

1. t is recommended that the authors define the full meaning of PDB, SDF, and PDBQT at the point of first use and for all the abbreviations. 
**Respond: we already define the full meaning of PDB, SDF, PSBQT and all abbreviations in our article.**
2. What is the full meaning of ADMET? Please provide the meaning at the time of first use.
**Respond: We think, we already mention full meaning ADMET in the abstract and introductions.** 
3. The authors should explain what type of interactions gallic acid and its derivatives make with the protein. Are they inhibitors or agonists? 
**Respond: we already explain the type of interaction gallic acid as agonis (page 7)**
4. In addition to discussing the relevance of the combined scores on the KEGG pathway, the authors should provide references to other studies related to this study. A description of the KEGG pathway is not sufficient. 
**Respond: We have added a discussion regarding the combined scores and other references related to the study (page 9)**
5. The authors are kindly requested to discuss the implications of this study for the different proteins and genes. What are the implications of this study for oncology? 
**Respond: in the end of discussion, we are adding our opinion about implication of this study for oncology (page 26)**
6. Molecular docking and ADMET profiling results should be discussed in detail, including the implications of each value for potential anticancer compounds of gallic and its derivatives.
**Respond: we think the explanation about ADMET has been fully explained in the article. Thank you.**
7. It is noteworthy that in-silico predictions are often not accurate, especially in clinical trials. In order to confirm the results obtained from in-silico screening, I recommend that the authors conduct some in-vitro admit profiling.
**Respond: While waiting for the review process for this article, we have conducted an in vitro test for this study. in this refinement article we have added relevant in vitro data and discussion.**
8. There is too much vagueness in the conclusion. A conclusion should be drawn based on the results of the study. 
**Respond:** **we have changed the conclusion of this article. Thank you.**

# Response

**Published:** 2023-08-30

**Authors:** Damilohun Samuel Metibemu

**Affiliations:** Biochemistry, Adekunle Ajasin University, Akungba, Ondo, Nigeria

Think the authors have responded well to my suggestions
